# Characterizing mitochondrial features in osteoarthritis through integrative multi-omics and machine learning analysis

**DOI:** 10.3389/fimmu.2024.1414301

**Published:** 2024-07-04

**Authors:** Yinteng Wu, Haifeng Hu, Tao Wang, Wenliang Guo, Shijian Zhao, Ruqiong Wei

**Affiliations:** ^1^ Department of Orthopedic and Trauma Surgery, the First Affiliated Hospital of Guangxi Medical University, Nanning, China; ^2^ Department of Orthopedics, Shandong Provincial Hospital Affiliated to Shandong First Medical University, Jinan, China; ^3^ Department of Orthopedic Joint, The First Affiliated Hospital of Guangxi Medical University, Nanning, China; ^4^ Department of Rehabilitation Medicine, the First Affiliated Hospital of Guangxi Medical University, Nanning, China; ^5^ Department of Cardiology, the Affiliated Cardiovascular Hospital of Kunming Medical University (Fuwai Yunnan Cardiovascular Hospital), Kunming, China

**Keywords:** osteoarthritis (OA), mitochondria, bulk RNA sequencing (bulk-RNA seq), single-cell RNA sequencing (scRNA-seq), immune cell infiltration

## Abstract

**Purpose:**

Osteoarthritis (OA) stands as the most prevalent joint disorder. Mitochondrial dysfunction has been linked to the pathogenesis of OA. The main goal of this study is to uncover the pivotal role of mitochondria in the mechanisms driving OA development.

**Materials and methods:**

We acquired seven bulk RNA-seq datasets from the Gene Expression Omnibus (GEO) database and examined the expression levels of differentially expressed genes related to mitochondria in OA. We utilized single-sample gene set enrichment analysis (ssGSEA), gene set enrichment analysis (GSEA), and weighted gene co-expression network analysis (WGCNA) analyses to explore the functional mechanisms associated with these genes. Seven machine learning algorithms were utilized to identify hub mitochondria-related genes and develop a predictive model. Further analyses included pathway enrichment, immune infiltration, gene-disease relationships, and mRNA-miRNA network construction based on these hub mitochondria-related genes. genome-wide association studies (GWAS) analysis was performed using the Gene Atlas database. GSEA, gene set variation analysis (GSVA), protein pathway analysis, and WGCNA were employed to investigate relevant pathways in subtypes. The Harmonizome database was employed to analyze the expression of hub mitochondria-related genes across various human tissues. Single-cell data analysis was conducted to examine patterns of gene expression distribution and pseudo-temporal changes. Additionally, The real-time polymerase chain reaction (RT-PCR) was used to validate the expression of these hub mitochondria-related genes.

**Results:**

In OA, the mitochondria-related pathway was significantly activated. Nine hub mitochondria-related genes (SIRT4, DNAJC15, NFS1, FKBP8, SLC25A37, CARS2, MTHFD2, ETFDH, and PDK4) were identified. They constructed predictive models with good ability to predict OA. These genes are primarily associated with macrophages. Unsupervised consensus clustering identified two mitochondria-associated isoforms that are primarily associated with metabolism. Single-cell analysis showed that they were all expressed in single cells and varied with cell differentiation. RT-PCR showed that they were all significantly expressed in OA.

**Conclusion:**

SIRT4, DNAJC15, NFS1, FKBP8, SLC25A37, CARS2, MTHFD2, ETFDH, and PDK4 are potential mitochondrial target genes for studying OA. The classification of mitochondria-associated isoforms could help to personalize treatment for OA patients.

## Introduction

Osteoarthritis (OA) is a chronic and disabling joint disease characterized predominantly by the progressive destruction of articular cartilage. It is the leading cause of declining quality of life among elderly individuals worldwide. According to the latest statistics from the World Health Organization, OA affects over 500 million people globally (1). The absence of a cure for OA can be attributed, in part, to an incomplete comprehension of the underlying pathological mechanisms governing its onset and progression. Consequently, it becomes imperative to enhance our understanding of the associated signaling pathways and critical molecules involved in OA development. This knowledge will facilitate the identification of therapeutic targets and aid in the design of effective treatments.

Mitochondria, as organelles within eukaryotic cells, produce adenosine triphosphate (ATP) through oxidative phosphorylation, thereby serving as the cell’s energy source (2). Additionally, they play a crucial role in cellular metabolism and maintaining cellular homeostasis. Impaired mitochondrial function reduces the capacity for oxidative phosphorylation, leading to an upsurge in reactive oxygen species (ROS) production. This accumulation of ROS can cause cellular damage or apoptosis (3). Mitochondria, as essential organelles in chondrocytes, play critical roles in cellular metabolism, proliferation, and apoptosis. Growing evidence suggests that mitochondrial dysfunction and disrupted energy metabolism are closely associated with the onset and progression of OA. However, the exact mechanisms remain unclear at present.

Research indicates that impaired mitochondrial function leads to increased levels of ROS, resulting in chondrocyte apoptosis and promoting the development of OA (4). It is noteworthy that oxidative stress is a key factor in causing mitochondrial DNA damage, impairing mitochondrial respiratory function, and activating mitochondria-mediated cell death pathways (5). Inflammatory cytokines like IL-1β and tumor necrosis factor-alpha (TNF-α) have been reported to reduce mitochondrial activity and ATP production, impairing mitochondrial respiration in chondrocytes and contributing to mitochondrial dysfunction in OA (6). Additionally, the balance between mitochondrial fission and fusion is essential for preserving proper mitochondrial function. Increased fission and decreased fusion can cause mitochondrial fragmentation and functional impairment, leading to reduced ATP production and increased ROS generation (7). Disrupted mitochondrial segregation during mitosis has also been implicated in mitochondrial dysfunction in OA (4). Additionally, alterations in mitochondrial metabolism may disrupt the cellular redox balance and contribute to the accumulation of ROS in OA. During the progression of OA, chondrocytes and synovial cells tend to shift their mitochondrial metabolism from oxidative phosphorylation to glycolysis, primarily regulated by the AMPK-MTOR pathway (8). Increasing evidence suggests that the loss of mitochondrial quality control homeostasis contributes to cartilage damage in the onset and progression of OA (9). Mitochondrial quality control is a crucial mechanism that coordinates various mitochondrial biological functions. Autophagy, a vital cellular mechanism for maintaining homeostasis, plays a critical role in chondrocytes and is closely linked to the pathogenesis of OA. Mitophagy, specifically, plays a crucial role in degrading, clearing, and recycling dysfunctional mitochondria, thus controlling mitochondrial quality and maintaining cellular homeostasis (10). Animal studies have confirmed that inhibiting mitophagy promotes cartilage degradation (11). Emerging evidence suggests that mitophagy plays a significant regulatory role in skeletal disorders, suggesting that modulating mitophagy levels could be a novel strategy for treating skeletal-related diseases (12). In summary, multiple factors, including oxidative stress, exacerbated inflammation, mitochondrial fission and fusion, disrupted mitosis, metabolic dysregulation in chondrocytes, and impaired autophagy, contribute to mitochondrial dysfunction in OA. Therefore, comprehensive understanding of the mechanisms by which mitochondrial dysfunction leads to the onset and progression of OA is crucial for developing effective strategies for its treatment.

By integrating bulk RNA, weighted gene co-expression network analysis (WGCNA), immune infiltration analysis, and molecular subtyping, we have investigated the potential alterations in mitochondria-related genes, their impact on pathways, and the role of immune cells. Additionally, we have identified targeted therapies, immune microenvironment, and pathway heterogeneity within different mitochondrial metabolic subgroups. Furthermore, we have developed and validated a predictive model for OA using machine learning and deep learning. Single-cell analysis revealed single-cell expression distribution patterns and putative temporal changes of hub mitochondria-related genes. Finally, real-time polymerase chain reaction (RT-PCR) verified the expression of hub mitochondria-related genes.

## Materials and methods

### Data acquisition and pre-processing

We retrieved OA-related chip datasets from the Gene Expression Omnibus (GEO) database (https://www.ncbi.nlm.nih.gov/geo/), which included GSE117999, GSE51588, GSE55235, GSE55457, GSE57218, GSE82107, and GSE98918. Mitochondria-related genes were obtained from the MitoCarta3.0 database. Overview of the information on analyzed datasets is provided in **Supplementary Table 1**. During the processing of the data, we excluded ineligible samples. During the processing of the chip datasets, we matched probe and gene names based on the annotation information of each GPL platform. In cases where multiple probes corresponded to the same gene, we selected the probe with the highest expression level for retention. Following this, we normalized the expression matrix using the `normalizeBetweenArrays` function and performed log2 transformation for datasets that required it. Next, we extracted the common genes shared by all seven datasets. To address batch effects and platform variations, we employed the ComBat method from the R package “sva” (13, 14) to normalize expression values across different batches or platforms. The success of batch effect removal was evaluated through principal component analysis (PCA).

### Differential expression analysis and enrichment analysis

Differential gene expression analysis was performed using the limma R package (15, 16) X to compare the expression profiles between OA samples and normal samples. The criteria for selecting differentially expressed genes (DEGs) associated with mitochondria were set as a p-value < 0.05. Next, we evaluated the activity scores of each pathway using the single-sample gene set enrichment analysis (ssGSEA) (17) and gene set enrichment analysis (GSEA) (18). Then, we utilized the clusterprofiler R package (19, 20) to perform functional and pathway analysis, including Gene Ontology (GO) (21) and Kyoto Encyclopedia of Genes and Genomes (KEGG), for the DEGs associated with mitochondria.

### WGCNA analysis

In the WGCNA analysis, we used the mitochondria-related DEGs as the background gene set. We calculated the Gene Set Variation Analysis (GSVA) (22) scores for each sample and used these GSVA scores as traits for the WGCNA analysis. On the basis of selecting all genes, the determination of the maximum R2 (power = 4) was conducted using the soft threshold of an unweighted network. Each module is required to contain at least 30 genes, and the topological overlap matrix similarity was utilized to evaluate the distance between gene pairs. Hierarchical clustering analysis was performed using the average linkage method and dynamic tree cut algorithm to construct a clustering tree and divide the genes into different modules. Next, we utilized the clusterprofiler R package (23) to perform GO and KEGG analysis, on the modules that were most correlated with the GSVA scores. String database to construct protein interaction networks for modular genes.

### Identification of hub mitochondria-associated DEGs

To identify hub genes among the mitochondria-related DEGs, we employed the Lasso regression, ridge regression, and elastic net regression for feature selection. Additionally, we utilized various methods such as SVM-RFE, Random Forest, Gradient Boosting Machine (GBM), and XGBoost to rank the importance of genes. We selected the top 30 genes based on their importance scores derived from these methods. The criteria for identifying hub genes among the mitochondria-related DEGs were set as the genes that appeared consistently across all seven algorithms mentioned above.

### Predictive model and alignment diagram

To determine the best machine learning model for predicting OA, we selected the expression profiles of hub mitochondria-associated DEGs as input variables, and built seven machine learning models including logistic regression, SVM, kknn, random forest, LDA, Naive Bayes, and decision tree for prediction using the “mlr3verse” R package. The reliability of the models was also verified using bootstrap method. The genes were then evaluated using multifactorial logistic regression analysis, and the area under the receiver operator characteristic (ROC) curve (AUC) was calculated using the “ROCR” package to assess the value of these genes in OA diagnosis. We used a nomogram to predict the likelihood of OA, and plotted correction curves and decision curves to analyze the stability of the model.

### Deep learning

Deep learning is a type of neural network that has more hidden layers and can understand more complex information structures in the data. Multi-Layer Perceptron (MLP) is a common form of neural network, also known as feedforward neural network. We compute the confusion matrix and visualize the MLP diagnostic results, extract the weights and biases of each layer, and finally generate the network structure and visualization.

### GSEA and ssGSEA analysis of hub mitochondria-associated DEGs

Using the mitochondrial gene set and KEGG pathway gene set as the background gene set, we performed GSEA analysis of hub mitochondria-related DEGs using the clusterprofiler R package. The ssGSEA algorithm was also utilized to calculate the score of the hallmark pathway set, and the correlation between the hub mitochondria-related DEGs and pathways was calculated using the spearman algorithm.

### Immune infiltration analysis

The cibersort (24) algorithm was utilized to calculate the proportion of immune cells in OA, and subsequently, the spearman algorithm was employed to assess the correlation between hub mitochondria-associated DEGs and immune cells. Additionally, the spearman algorithm was used to determine the correlation between hub mitochondria-associated DEGs and immune-related genes.

### Constructing gene-disease networks and mRNA-miRNA networks

The DisGeNET R package (25) was utilized to analyze diseases associated between genes, while multiple miRAN databases were integrated to predict the miRAN of genes.

### Genome−wide association study

The GeneAtlas database (http://geneatlas.roslin.ed.ac.uk/) serves as a comprehensive resource that utilizes data from the UK Biobank cohort to provide extensive information on associations between hundreds of traits and millions of genetic variations. The database contains information from the UK Biobank, including data from 452,264 individuals. It covers 778 phenotypes and encompasses a vast number of genetic loci, totaling approximately 30 million. It offers valuable genetic and phenotypic data to researchers, facilitating a deeper understanding of the relationships between genes and traits and advancing research in related fields.

### Consensus clustering

Consensus clustering (26, 27) is a comprehensive clustering method designed to identify subgroups within a dataset. In this study, we performed clustering analysis using the K-means algorithm on the expression profiles of mitochondria-related DEGs in OA samples. We specified a maximum of 10 clusters and determined the final number of clusters based on the consistency matrix and cluster consensus score, which exceeded 0.8. Subsequently, we further analyzed the differential expression levels of the mitochondria-related DEGs and immune cells across different subgroups.

### Drug analysis

Utilizing the results from the subtype differential expression as disease indicators and the drug data in the CMap database as drug markers, we implemented the eXtreme Sum (XSum) methodology for feature alignment. This was done to identify drugs that are suitable for particular subtypes.

### GSEA, GSVA and protein function analysis of subtypes

To look for subtype-related fe and dynamic tree-cutting methods. This approach facilitated the creation of a dendrogram and the assignment of genes to their respective modules. After considering their similarities, we merged the original 11 modules and ultimately identified six functional modules. To further analyze the key modules, we utilized the ClusterProfiler R package to perform GO and KEGG functional analyses.

### Analysis of expression levels in normal human tissues

Expression of hub mitochondria-related genes in human tissues was analyzed using the harmonizome database (https://maayanlab.cloud/Harmonizome/).

### Single-cell analysis

We used the GSE133449 dataset for single-cell level expression distribution and pseudotime analysis. We processed the single-cell RNA sequencing data using the Seurat R package (28). Cells expressing more than 200 but not exceeding 2,500 genes were selected. The “FindVariableGenes” function was employed to identify highly variable genes, followed by PCA. For dimensionality reduction and visualization of the single-cell data, we utilized Uniform Manifold Approximation and Projection (UMAP). To generate a plot of the single-cell data, we used the “DimPlot” function, and for visualizing gene expression patterns, we employed the “FeaturePlot” function. Cell type annotation was performed using the ‘HumanPrimaryCellAtlasData()’ function from the ‘celldex’ package. Subsequently, we calculated senescence scores for each cell type and evaluated differences in senescence scores between the normal and OA groups for the same cell types. Further visualization of these differences can be achieved using the “FeaturePlot” function. Note: ‘HumanPrimaryCellAtlasData()’ is not actually a function in the ‘celldex’ package. It is used here as an example for cell type annotation. Please refer to the appropriate package or resource for cell type annotation in your specific analysis.

### Pseudotime analysis

We extracted a subset of chondrocyte cells from the single-cell data for pseudotime analysis. Initially, we performed re-dimensionality reduction and clustering specifically on the chondrocyte cells. Next, we utilized the Monocle R package for pseudotime analysis. We selected cells for subsequent pseudotime analysis based on average expression greater than 0.1 and empirical dispersion greater than one-fold of the fitted dispersion. To reduce dimensionality and order the cells in the pseudotime analysis, we used the ‘DDRTree’ method from the ‘reduceDimension’ function. Additionally, We examined the expression variations of mitochondria-related genes across distinct clusters during the cell differentiation trajectory.

### RT-PCR validation

Human chondrocyte cells were obtained from Wuhan Saos Technology Co., Ltd. These cells were cultured in DMEM/F12 medium supplemented with 10% fetal bovine serum. To induce inflammation, the model group’s cells were treated with a culture medium containing IL-1β at a concentration of 10 ng/ml for 24 hours. Total RNA extraction was performed using the QIAzol reagent kit, followed by cDNA synthesis through reverse transcription using oligo-dT primers. Subsequently, gene amplification of the cDNA was carried out using an RT-PCR machine, following an initial denaturation at 95°C for 5 minutes, followed by 40 cycles of denaturation at 95°C for 1 minute, annealing at the optimal temperature of 60°C for 30 seconds, and extension at 72°C for 1 minute. The relative expression level of each specific gene was calculated using the 2-ΔΔ method. Please refer to **Table 1** for the primer sequences of the human genes utilized.

**Table 1:** Primers used in this study.

Primer	Sequence
SLC25A37-F	AGAAAATCATGCGGACCGAAG
SLC25A37-R	TGGTGGTGGAAAACGTCATTTA
MTHFD2-F	AGGACGAATGTGTTTGGATCAG
MTHFD2-R	GGAATGCCAGTTCGCTTGATTA
SIRT4-F	GCTTTGCGTTGACTTTCAGGT
SIRT4-R	CCAATGGAGGCTTTCGAGCA
DNAJC15-F	TTGCAGGTCGCTACGCATTT
DNAJC15-R	CCAGCTTCTCGCCTACTCATT
ETFDH-F	TACTGTGCCTCGAATTACTACCC
ETFDH-R	ACAGCCAACTGTTTTAGACGAA
PDK4-F	GGAAGCATTGATCCTAACTGTGA
PDK4-R	GGTGAGAAGGAACATACACGATG
CARS2-F	AAGCCGCCTCCTGGTATAG
CARS2-R	CTTCCTCATAAAGACTGGCGAG
FKBP8-F	GACTTCGAGGTACTGGATGGG
FKBP8-R	CTTCTTCCTCAACAGCCCGTT
NFS1-F	TGGATGTGCAAGCTACAACTC
NFS1-R	GATCAGCTCCAATCAGAGATGC
GAPDH-F	TCAAGATCATCAGCAATGCC
GAPDH-R	CGATACCAAAGTTGTCATGGA

## Results

### Data processing and enrichment analysis

**Figure 1:**
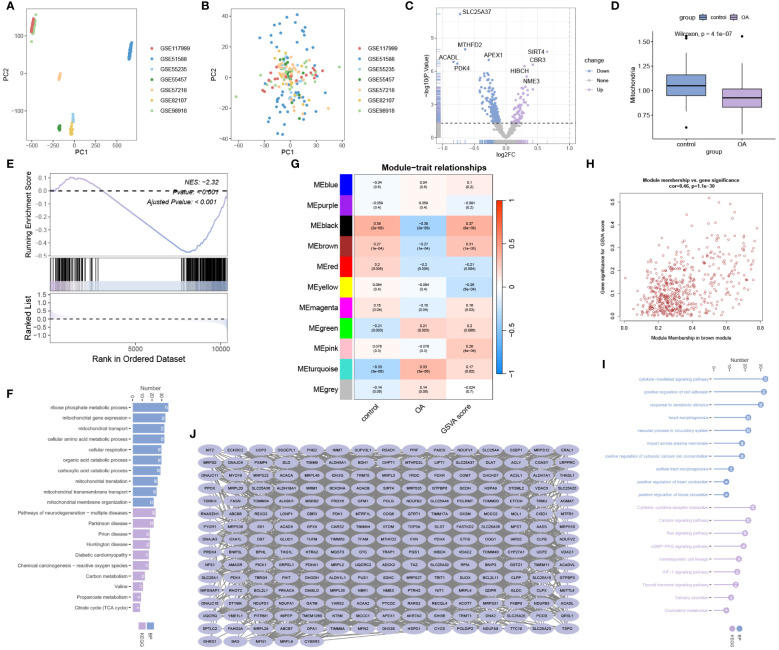
Data processing and enrichment analysis. **(A)** Data before batch effect removal. **(B)** Data after batch effect removal. **(C)** Volcano plot for differential expression analysis. **(D)** ssGSEA analysis and **(E)** GSEA analysis reveal a significant decrease in mitochondrial activity in OA. **(F)** Gene Ontology Biological Process (BP) and Kyoto Encyclopedia of Genes and Genomes (KEGG) analysis for mitochondria-related DEGs. **(G)** WGCNA analysis for mitochondrial scoring. **(H)** Module Membership (MM) and Gene Significance (GS) values for genes in the black module. **(I)** Gene Ontology BP and KEGG analysis for genes in the black module. **(J)** Protein-protein interaction network analysis for genes in the black module.

We integrated and processed data from GSE117999, GSE51588, GSE55235, GSE55457, GSE57218, GSE82107, and GSE98918, resulting in 66 control samples and 125 OA samples, encompassing information on 10827 genes. As depicted in **Figure 1A**, batch effects were observed across the seven different datasets. However, after eliminating these batch effects, consistent results were obtained (**Figure 1B**). This indicates that we successfully eliminated batch effects through cross-platform standardization methods. Differential expression analysis revealed 245 mitochondria-related DEGs, including 72 up-regulated and 173 down-regulated mitochondria-related DEGs (**Figure 1C**). The ssGSEA results demonstrated a significant decrease in mitochondrial activity in OA (**Figure 1D**), which was further validated by GSEA analysis (**Figure 1E**). Enrichment analysis revealed significant enrichment of mitochondria-related DEGs in processes such as ribose phosphate metabolic process, mitochondrial gene expression, mitochondrial transport, Pathways of neurodegeneration - multiple diseases, Parkinson disease, and Prion disease (**Figure 1F**). WGCNA analysis indicated a significant correlation between the black module and mitochondrial scores (**Figure 1G**). **Figure 1H** displays the Module Membership (MM) and Gene Significance (GS) for each gene in the black module. Both BP and KEGG analyses showed significant enrichment of genes in the black module in processes such as cytokine-mediated signaling pathway, positive regulation of cell adhesion, response to xenobiotic stimulus, Cytokine-cytokine receptor interaction, Calcium signaling pathway, and Ras signaling pathway (**Figure 1I**). The protein-protein interaction network of genes in the black module is illustrated in **Figure 1J**.

### Machine learning identification of hub mitochondria-related DEGs

**Figure 2:**
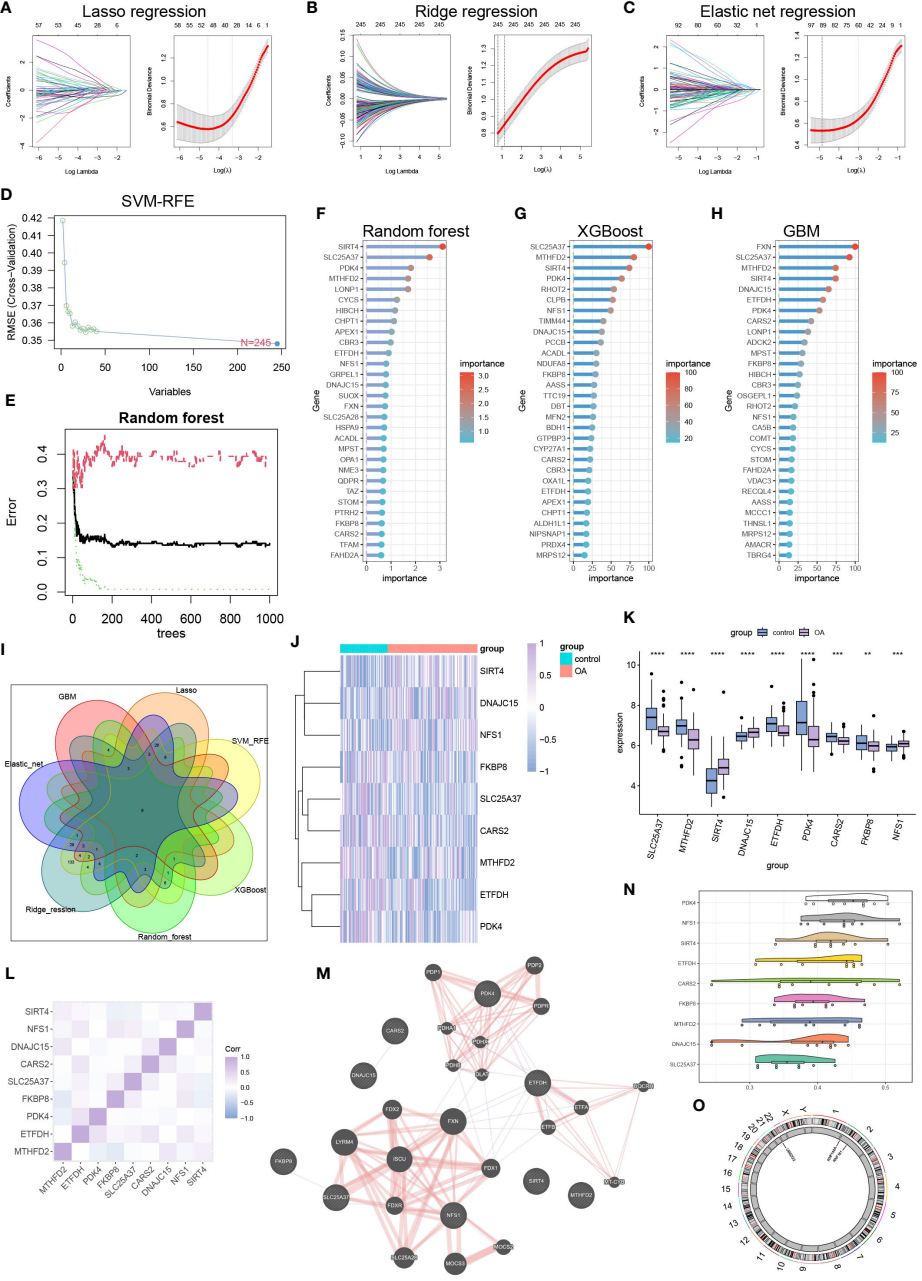
Machine learning to identify hub mitochondria-associated DEGs. **(A)** Lasso regression, **(B)** Ridge regression, **(C)** Elastic Net regression, and **(D)** SVM-RFE were utilized to select mitochondria-related DEGs. **(E, F)** Random Forest analysis, **(G)** Xgboost analysis, and **(H)** GBM analysis identified the top 30 mitochondria-related DEGs. **(I)** A Venn diagram illustrated that nine hub mitochondria-related DEGs were identified by the seven machine learning methods. Heatmap **(J)** and boxplot **(K)** displayed the expression levels of these nine hub mitochondria-related DEGs (**p < 0.01, ***p < 0.001, ****p < 0.0001). Correlation analysis **(L)**, protein-protein interaction analysis **(M)**, functional similarity analysis **(N)**, and chromosomal loci information analysis **(O)** were conducted for the hub mitochondria-related DEGs.

We successfully utilized lasso regression (**Figure 2A**), ridge regression (**Figure 2B**), and elastic regression (**Figure 2C**) to screen for important mitochondria-related DEGs. Additionally, we employed Support Vector Machine - Recursive Feature Elimination (SVM-RFE) to select the top 30 important mitochondria-related DEGs (**Figure 2D**). Moreover, we used random forest (**Figures 2E, F**), Xgboost (**Figure 2G**), and GBM (**Figure 2H**) to independently identify the top 30 mitochondria-related DEGs. Subsequently, we obtained the intersection of these seven machine learning results, resulting in nine hub mitochondria-related DEGs, namely SLC25A37, MTHFD2, SIRT4, DNAJC15, ETFDH, PDK4, CARS2, FKBP8, and NFS1 (**Figure 2I**). The heatmap displayed the expression patterns of SLC25A37, MTHFD2, SIRT4, DNAJC15, ETFDH, PDK4, CARS2, FKBP8, and NFS1 in OA and control samples (**Figure 2J**). Box plots illustrated the expression levels of these genes in OA and control samples (**Figure 2K**). The correlation and protein-protein interaction information of these genes are presented in **Figures 2L** and **M**, respectively. Functional similarity analysis revealed that PDK1 holds a significant position among these genes (**Figure 2N**). The chromosomal locations of these genes are shown in **Figure 2O**.

### Column chart and neural network model construction

**Figure 3:**
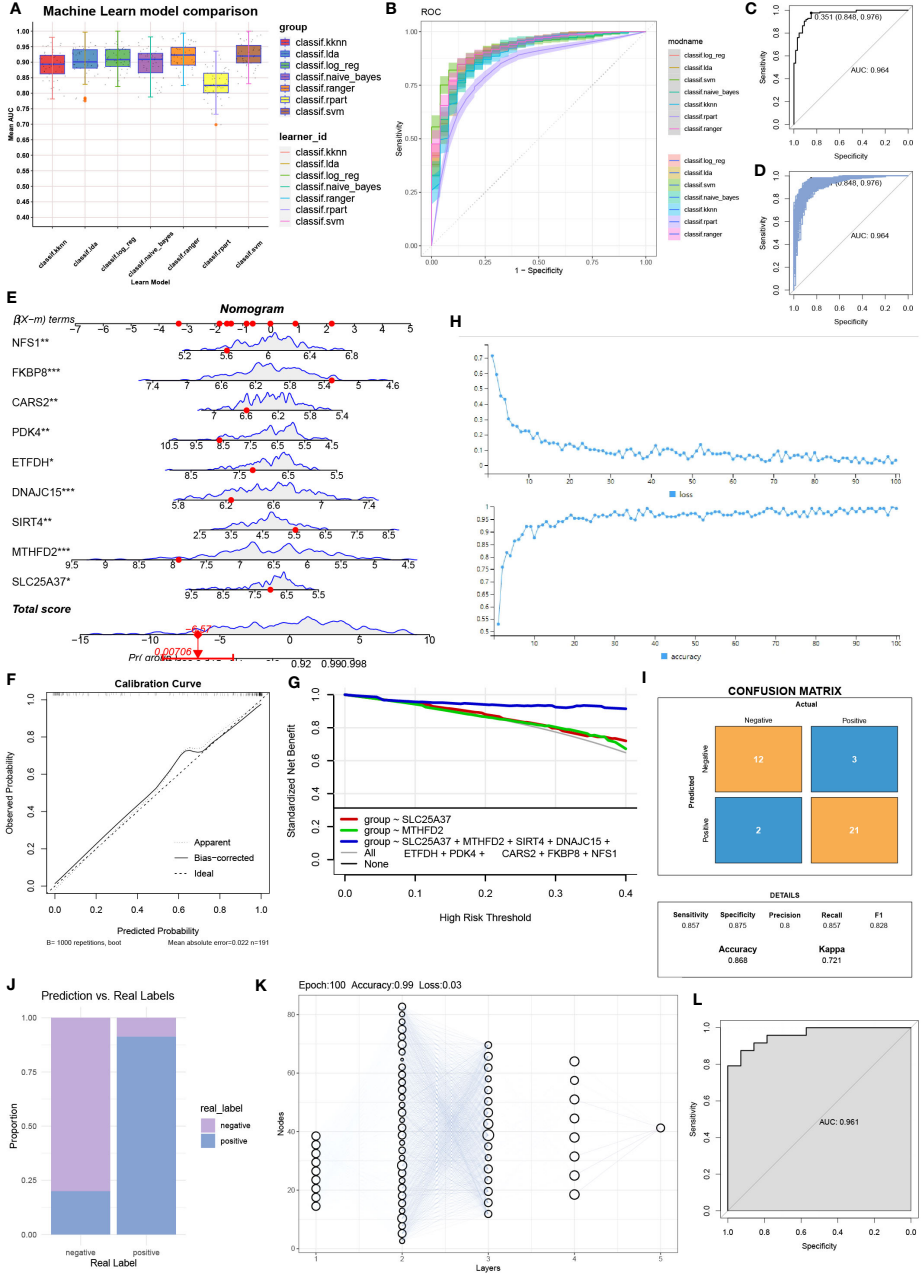
Column line graphs and neural network building. **(A)** Comparison of the AUC (Area Under the Curve) for the seven machine learning models. **(B)** Comparison of the ROC (Receiver Operating Characteristic) curves for the seven machine learning models. **(C)** AUC for the random forest model and **(D)** internal validation. **(E)** Detailed information in column plot format. **(F)** Calibration curve for the diagnostic model. **(G)** Model evaluation curves. **(H)** Accuracy and loss obtained at different iteration counts during model training. **(I)** Confusion matrix. **(J)** Actual prediction capability. **(K)** Structure of the neural network. **(L)** Prediction effect of the deep learning model constructed.

After comparing seven machine learning models, we found that the random forest model exhibited the highest AUC value (**Figure 3A**). Additionally, the random forest model demonstrated good sensitivity and specificity (**Figure 3B**). The AUC of the random forest model was 0.968 (**Figure 3C**), indicating its ability to accurately identify OA patients. Internal validation further confirmed the reliability of the model (**Figure 3D**). In the column chart, each feature variable corresponds to a specific score, and the sum of all feature scores represents the probability of having OA (**Figure 3E**). The calibration curve validated the accuracy of the column chart in diagnosing OA (**Figure 3F**). Decision Curve Analysis (DCA) showed that the column chart provided certain benefits for OA patients in clinical applications (**Figure 3G**). During the construction of the neural network model, **Figure 3H** displayed the accuracy and loss rate for each of the 100 training iterations. As indicated by the Kappa test (**Figure 3I**), the confusion matrix exhibited good consistency. **Figure 3J** presented a comparison between the predicted and actual results. The structure of the neural network model is depicted in **Figure 3K**. The model achieved an AUC of 0.961, demonstrating high diagnostic performance (**Figure 3L**).

### Enrichment analysis of hub mitochondria-related DEGs

**Figure 4:**
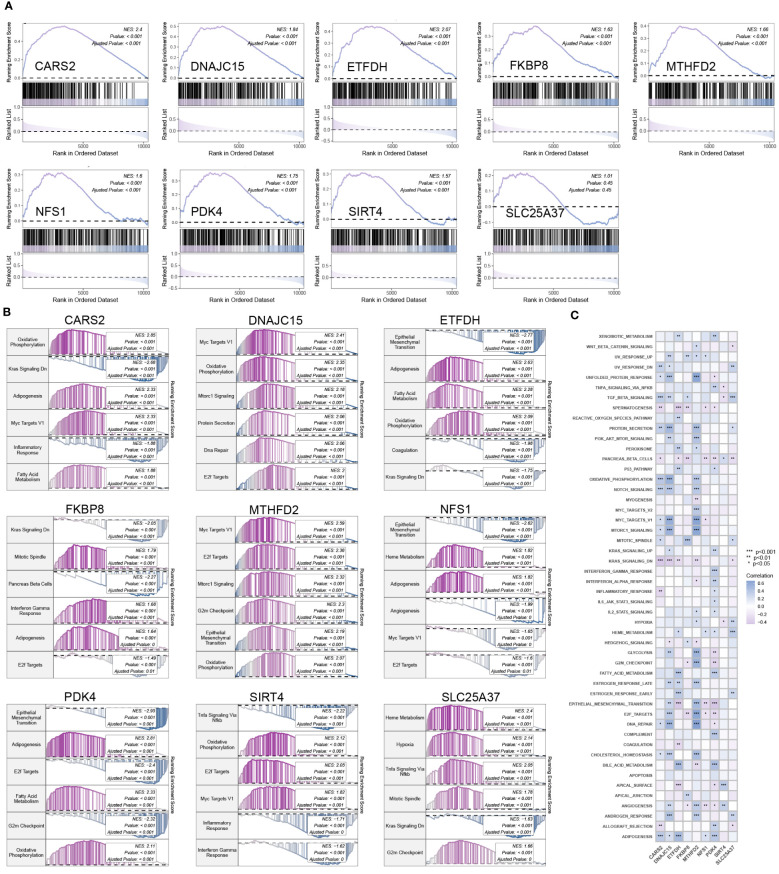
Enrichment analysis of hub mitochondria-associated DEGs. **(A)** GSEA analysis for SLC25A37, MTHFD2, SIRT4, DNAJC15, ETFDH, PDK4, CARS2, FKBP8, and NFS1 using the mitochondria gene set as the background gene set. **(B)** GSEA analysis for SLC25A37, MTHFD2, SIRT4, DNAJC15, ETFDH, PDK4, CARS2, FKBP8, and NFS1 using the KEGG pathway gene set as the background gene set. **(C)** ssGSEA analysis for SLC25A37, MTHFD2, SIRT4, DNAJC15, ETFDH, PDK4, CARS2, FKBP8, and NFS1 using the hallmark pathway gene set as the background gene set.

Except for SLC25A37, MTHFD2, SIRT4, DNAJC15, ETFDH, PDK4, CARS2, FKBP8, and NFS1 were significantly activated in mitochondrial pathways (**Figure 4A**). **Figure 4B** provides detailed information on the top six pathways that showed significant enrichment when using the KEGG pathway gene set as the background gene set for hub mitochondria-related DEGs. **Figure 4C** illustrates the correlation between hub mitochondria-related DEGs and hallmark pathway gene sets.

### Immune infiltration analysis

**Figure 5:**
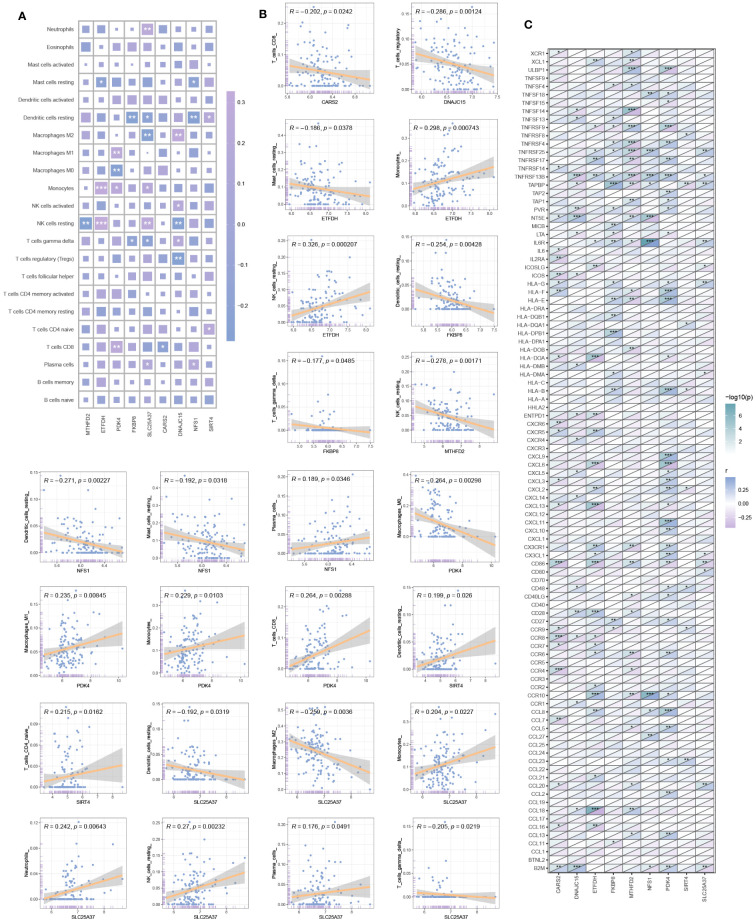
Immune infiltration analysis. **(A)** Correlation heatmap between hub mitochondria-related DEGs and immune cells. **(B)** Scatter plot detailing the correlation between each hub mitochondria-related DEGs and each immune cell. **(C)** Correlation between hub mitochondria-related DEGs and immune-related genes (*p < 0.05, **p < 0.01, ***p < 0.001).

The heatmap in **Figure 5A** depicts the correlation between hub mitochondria-related DEGs and immune cells. Subsequent selection of results with significant correlations is presented in the scatter plot, providing a detailed visualization of the correlation between each hub mitochondria-related DEG and individual immune cells (**Figure 5B**). SIRT4 exhibits positive correlation with Dendritic cells resting and T cells CD4 naive. DNAJC15 displays positive correlation with Macrophages M2, NK cells activated, and T cells gamma delta, while exhibiting negative correlation with NK cells resting and T cells regulatory (Tregs). NFS1 demonstrates positive correlation with Plasma cells and negative correlation with Mast cells resting and Dendritic cells resting. FKBP8 exhibits negative correlation with Dendritic cells resting and T cells gamma delta. SLC25A37 shows positive correlation with Neutrophils, Monocytes, NK cells resting, and Plasma cells, while demonstrating negative correlation with Dendritic cells resting, Macrophages M2, and T cells gamma delta. CARS2 exhibits negative correlation with T cells CD8. MTHFD2 shows negative correlation with NK cells resting. ETFDH displays positive correlation with Monocytes and NK cells resting, while showing negative correlation with Mast cells resting. PDK4 demonstrates negative correlation with Macrophages M1, Monocytes, and T cells CD8, while displaying negative correlation with Macrophages MO. Further analysis reveals the correlation between hub mitochondria-related DEGs and immune-related genes, as depicted in **Figure 5C**.

### Gene-disease network and mRNA-miRNA network

**Figure 6:**
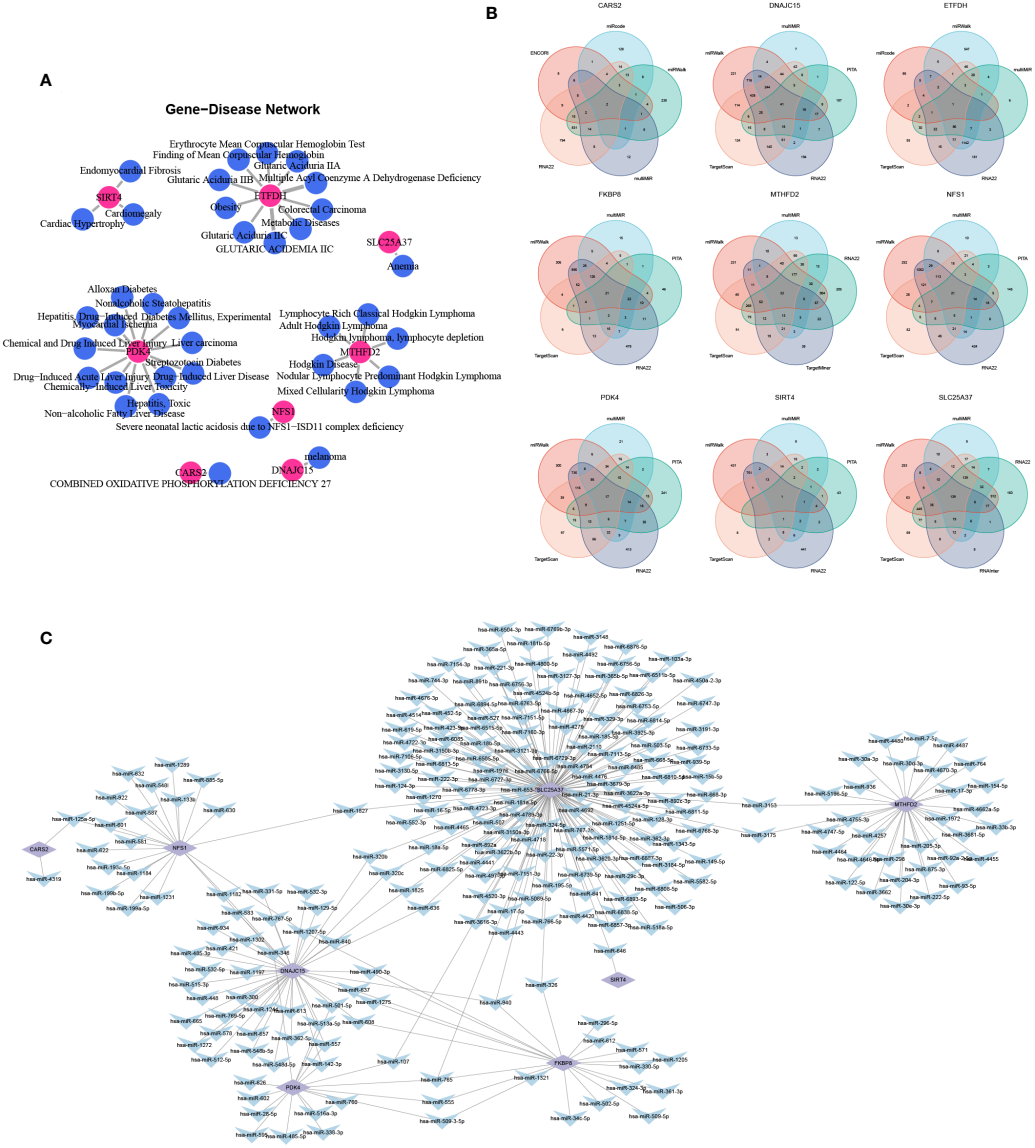
Gene-disease networks and mRNA-miRNA networks. **(A)** Gene-disease network (red circles represent hub mitochondria-related genes; blue circles represent diseases). **(B)** Venn diagram displaying the miRNA sets for each hub mitochondria-related DEGs. **(C)** mRNA-miRNA network (purple shapes represent hub mitochondria-related genes; blue shapes represent miRNAs).

By analyzing the associations between genes and diseases, we constructed a gene-disease network (**Figure 6A**). For hub mitochondria-related DEGs, we selected five databases that encompassed the maximum number of miRNAs (**Figure 6B**) and successfully constructed an mRNA-miRNA network (**Figure 6C**).

### GWAS analysis of hub mitochondria-related genes

**Figure 7:**
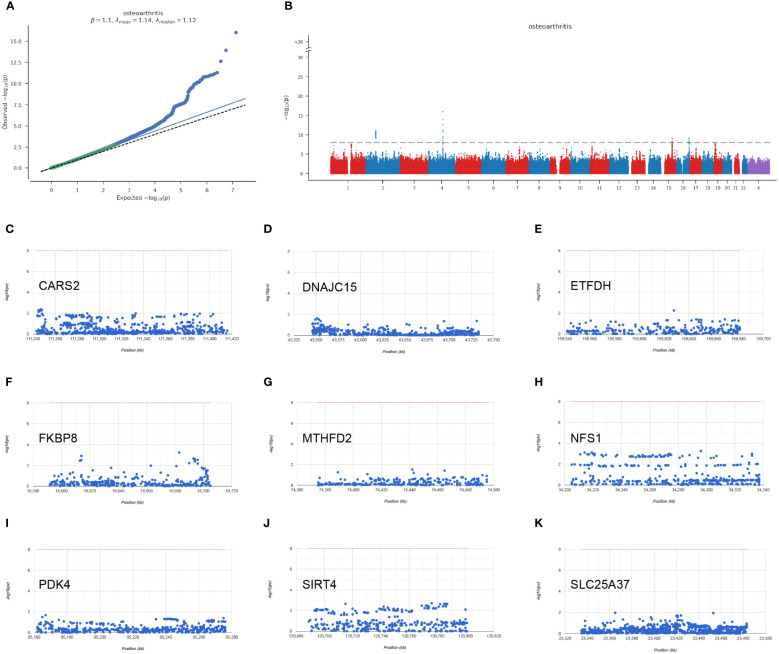
GWAS analysis results. **(A)** Q-Q plot for GWAS (Genome-Wide Association Study). **(B)** Manhattan plot for GWAS. **(C–K)** Chromosomal regions of CARS2, DNAJC15, ETFDH, FKBP8, MTHFD2, NFS1, PDK4, SIRT4, SLC25A37.

Through the analysis of GWAS data, we have identified the disease-associated regions for eight hub mitochondria-related genes in OA (**Figures 7A, B**). The plot also displays the single nucleotide polymorphism (SNP) disease-associated regions corresponding to CARS2, DNAJC15, ETFDH, FKBP8, MTHFD2, NFS1, PDK4, SIRT4, and SLC25A37 (**Figures 7C–K**).

### Consensus clustering analysis

**Figure 8:**
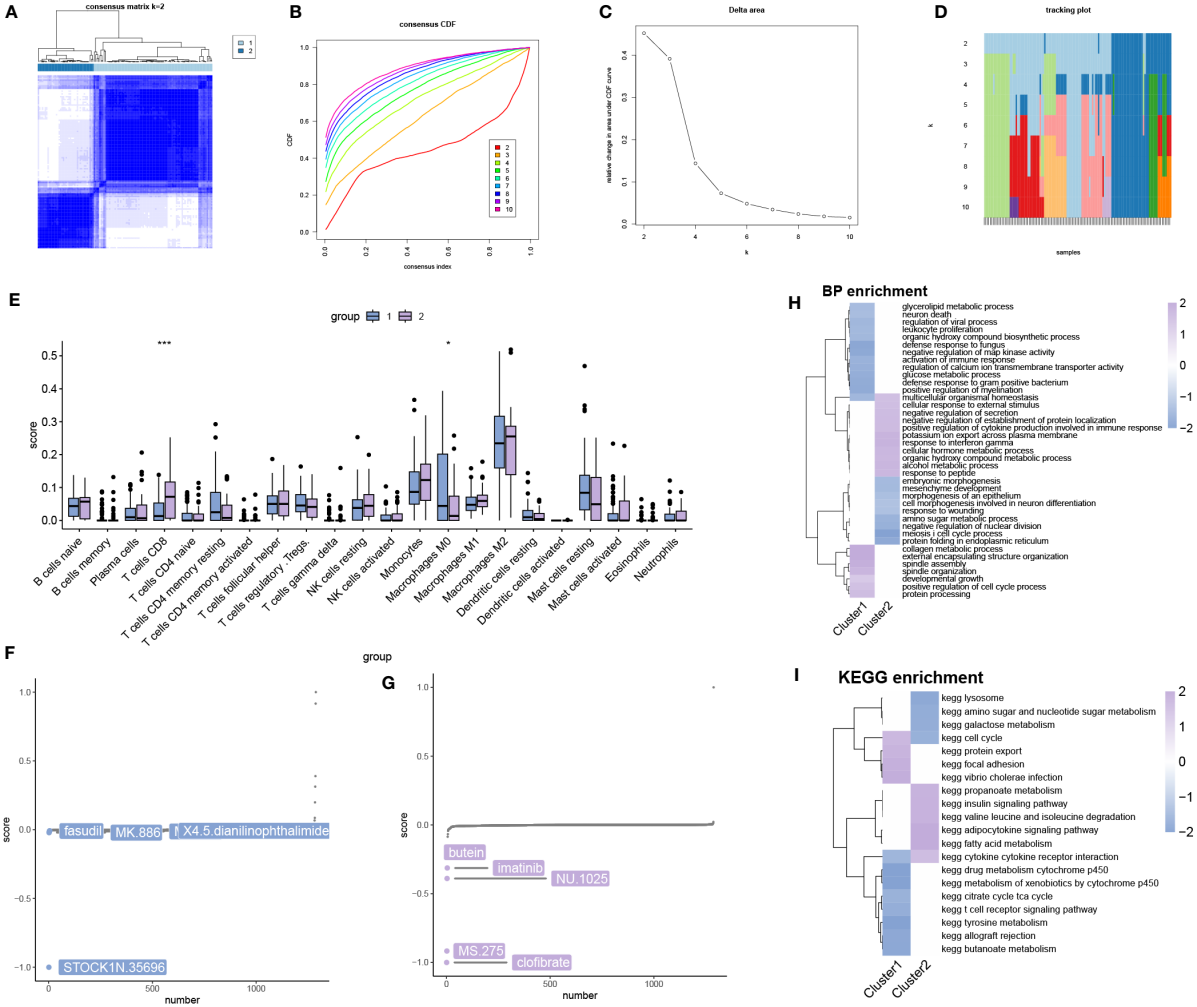
Identification of molecular clusters and characterization of subtypes based on hub mitochondria-related DEGs in OA. **(A)** Consensus clustering matrix for k = 2. **(B)** Cumulative distribution function (CDF) curves for clustering. **(C)** CDF delta area curves. **(D)** Tracking plot. **(E)** Differential analysis of immune cells between subtype 1 and subtype 2 (*p < 0.05, ***p < 0.0001). Drug analysis results for treating patients of subtype 1 **(F)** and subtype 2 **(G)**. GSEA analysis between subtypes, including BP **(H)** and KEGG **(I)** levels.

We performed classification of OA samples based on hub mitochondria-related DEGs. Using the consensus matrix as a similarity matrix, we determined the final subtypes. Based on the consensus clustering results, cumulative distribution function (CDF) plot, relative changes in CDF curves, and consensus clustering scores, we selected k=2 as the optimal value, dividing the OA samples into two distinct subtypes (**Figures 8A–D**). In subtype 1, CD8 T cells showed significantly lower expression, while M0 macrophages exhibited significantly higher expression (**Figure 8E**). Drug analysis revealed that STOCK1N.35696, fasudil, MK.886, and X4.5.dianilinophthalimide were among the top five potential drugs for treating subtype 1 patients (**Figure 8F**), whereas clofibrate, MS.275, NU.1025, imatinib, and butein were among the top five applicable drugs for treating subtype 2 patients (**Figure 8G**). **Figures 8H** and **8I** respectively illustrate some biological processes and KEGG pathways involved in the subtypes.

### Protein-level enrichment analysis and GSVA analysis of Subtypes

**Figure 9:**
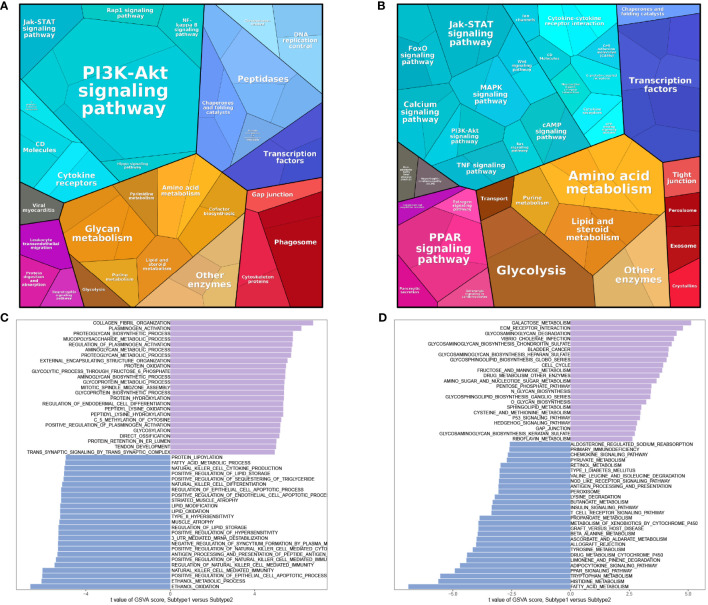
Enrichment analysis of subtypes. Protein pathway analysis of subtype 1 **(A)** and subtype 2 **(B)**. GSVA analysis of BP **(C)** and KEGG **(D)**.

Protein pathway analysis revealed that subtype 1 was primarily enriched in the PI3K-Akt signaling pathway (**Figure 9A**), while subtype 2 showed enrichment in transcription factors (**Figure 9B**). GSVA analysis indicated that both subtype 1 and subtype 2 were associated with numerous metabolic functions or pathways in terms of biological processes (BP) (**Figure 9C**) and KEGG pathways (**Figure 9D**).

### WGCNA analysis of subtypes

**Figure 10:**
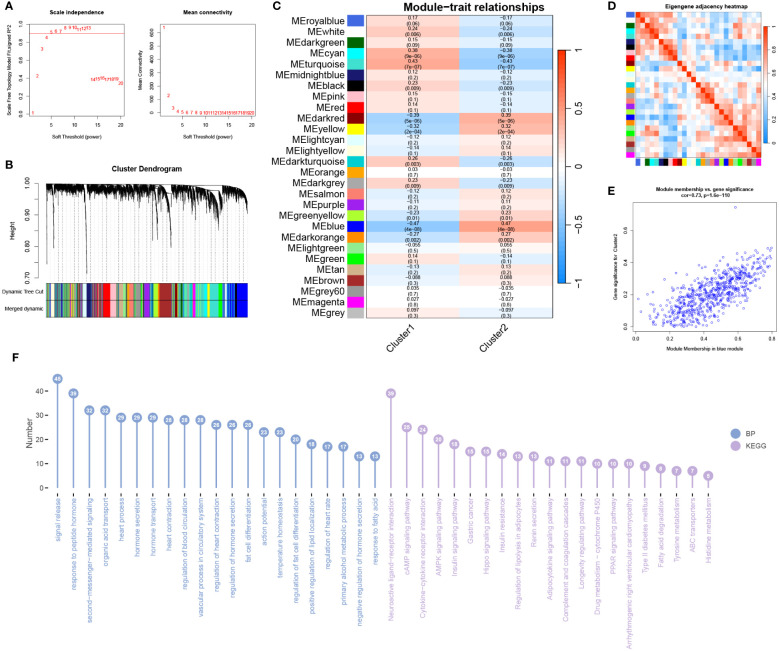
WGCNA analysis of subtypes. **(A)** The optimal soft threshold value is determined to be 4. **(B)** Merged modules after merging process. **(C)** Correlation analysis between modules and traits. **(D)** Heatmap showing the correlation between modules. **(E)** Scatter plot displaying Module Membership (MM) and Gene Significance (GS) for genes in the grey60 module. **(F)** BP and KEGG analysis for genes in the module.

Using the information from subtype 1 and subtype 2, we conducted WGCNA analysis. The soft-thresholding power was set to 4 (**Figure 10A**), with a correlation coefficient threshold of 9. This resulted in the generation of 27 modules (**Figure 10B**). Among these modules, the blue module exhibited the strongest correlation with the subtypes (**Figure 10C**). Inter-module correlations are illustrated in **Figure 10D**. Module-trait analysis focused on the blue module, displaying the distribution of MM and GS for genes within this module (**Figure 10E**). Regarding BP, enrichment within the module primarily involved signal release, response to peptide hormone, and second-messenger-mediated signaling (**Figure 10F**). In terms of KEGG pathways, the module genes were predominantly enriched in Neuroactive ligand-receptor interaction, cAMP signaling pathway, and Cytokine-cytokine receptor interaction (**Figure 10F**).

### Human tissue expression atlas

**Figure 11:**
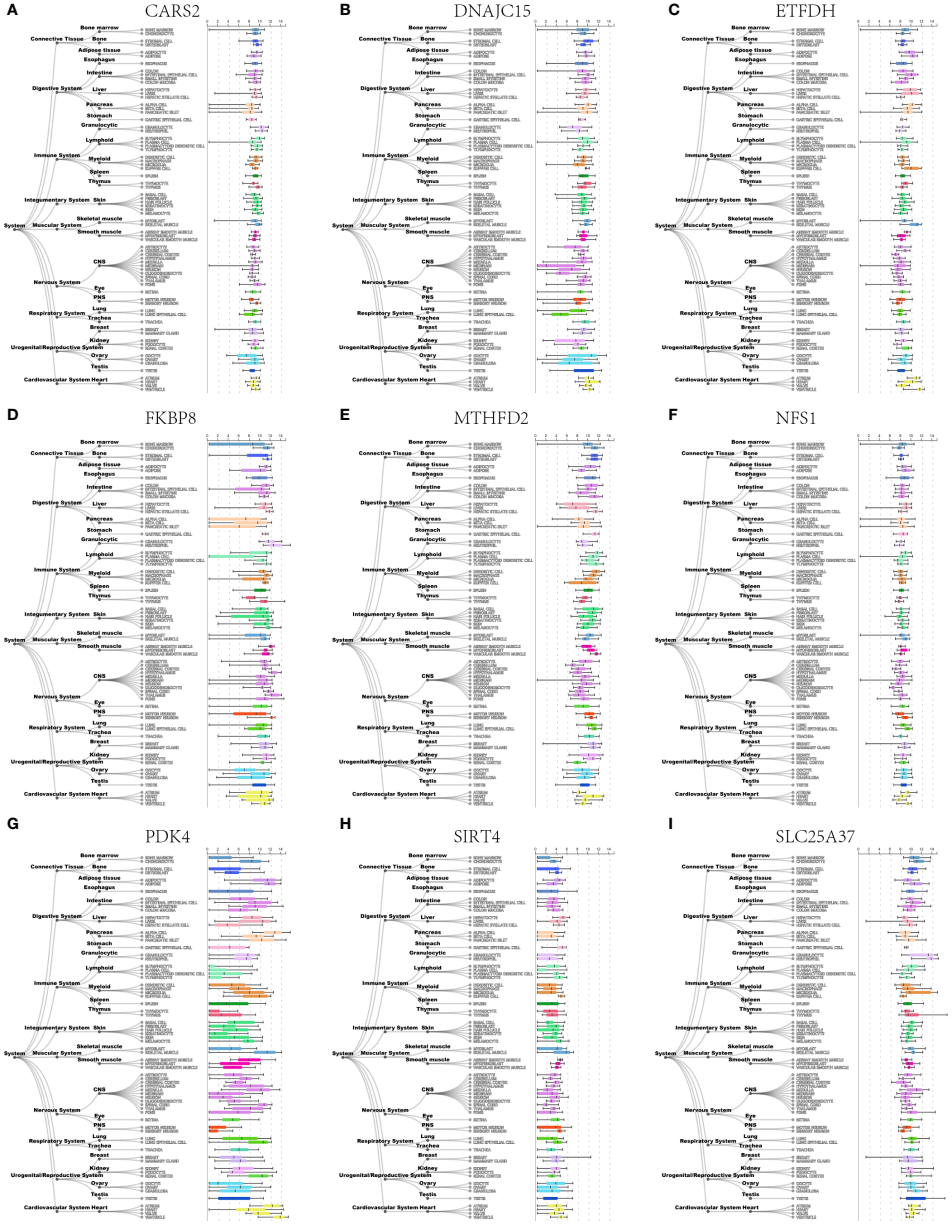
Expression profiles of nine hub mitochondria-related genes, including CARS2 **(A)**, DNAJC15 **(B)**, ETFDH **(C)**, FKBP8 **(D)**, MTHFD2 **(E)**, NFS1 **(F)**, PDK4 **(G)**, SIRT4 **(H)**, and SLC25A37 **(I)** in human tissues.

Utilizing the Harmonizome database, we analyzed the expression of hub mitochondria-related DEGs in human tissues (**Figures 11A–I**), with a particular focus on bone and cartilage cells.

### Single-cell expression and pseudotime analysis

**Figure 12:**
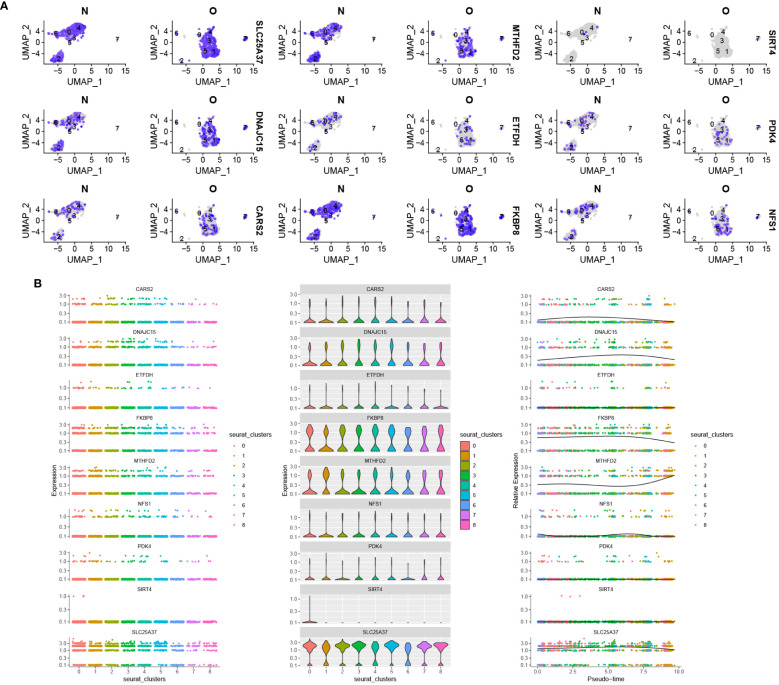
Distribution of hub mitochondria-related genes in OA based on single-cell RNA sequencing data. **(A)** Violin plot showing the distribution of 9 hub mitochondria-related genes in different cell types. **(B)** The 9 hub mitochondria-related genes produced expression changes in the proposed time series.

**Figure 12A** depicts the distribution and expression of hub mitochondria-related genes in different cell types. We found that SIRT4, DNAJC15, NFS1, FKBP8, SLC25A37, CARS2, MTHFD2, ETFDH, and PDK4 were expressed in single cells. During the process of cell differentiation, the expression patterns of SIRT4, DNAJC15, NFS1, FKBP8, SLC25A37, CARS2, MTHFD2, ETFDH, and PDK4 underwent changes (**Figure 12B**).

### RT-PCR validation results

**Figure 13:**
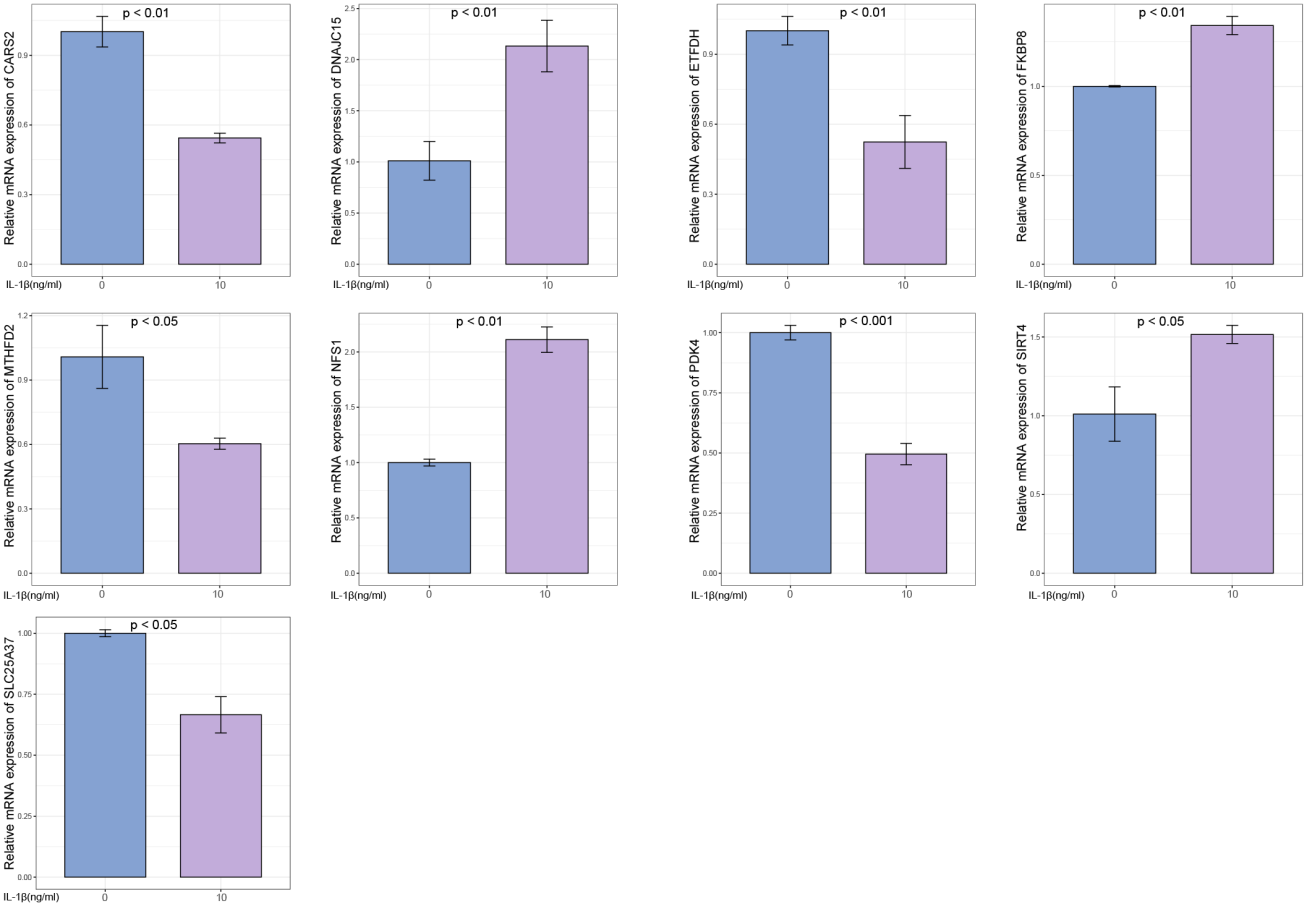
Experimental validation of key gene expression. Relative mRNA expression of SIRT4, DNAJC15, NFS1, FKBP8, SLC25A37, CARS2, MTHFD2, ETFDH, and PDK4.

The OA group exhibited higher expression levels of DNAJC15, FKBP8, NFS1, and SIRT4 genes compared to the control group. Conversely, the OA group showed lower expression levels of CARS2, ETFDH, MTHFD2, PDK4, and SLC25A37 genes in comparison to the control group (**Figure 13**).

## Discussion

OA is typically caused by abnormal metabolism and death of chondrocytes. The functionality of mitochondria is crucial for maintaining a stable intracellular environment in chondrocytes. Impaired mitochondrial function, a significant factor in cellular metabolic abnormalities, contributes to the onset and progression of OA. Recent studies have also reported that dysfunctional mitochondria in chondrocytes can lead to the generation of ROS, oxidative stress, inflammation, and cell apoptosis (29). Therefore, alterations in mitochondrial function are associated with the pathological processes of OA.

In this study, we systematically analyzed genes associated with mitochondrial metabolism in OA. Through differential expression analysis, we identified a set of differentially expressed genes linked to mitochondrial metabolism. Furthermore, employing the SsGSEA algorithm, we evaluated the expression levels of the mitochondrial metabolism-related gene set and observed significant downregulation in OA. This finding highlights the influence of mitochondrial metabolic dysfunction on the development of OA. Through enrichment analysis, we discovered that the main biological processes involving mitochondrial metabolism-related genes included mitochondrial gene expression, mitochondrial transport, mitochondrial translation, mitochondrial transmembrane transport, and mitochondrial membrane organization. Furthermore, we identified significant signaling pathways associated with mitochondrial metabolism-related genes, including cytokine-mediated signaling pathway, positive regulation of cell adhesion, response to xenobiotic stimulus, cytokine-cytokine receptor interaction, calcium signaling pathway, Ras signaling pathway, cGMP-PKG signaling pathway, hematopoietic cell lineage, and HIF-1 signaling pathway. These findings serve as a theoretical foundation for further exploring the underlying mechanisms of mitochondrial metabolism in OA.

Through the use of seven machine learning algorithms, we identified nine mitochondrial metabolism-related genes, namely SIRT4, DNAJC15, NFS1, FKBP8, SLC25A37, CARS2, MTHFD2, ETFDH, and PDK4, which are closely associated with the occurrence of OA. Based on these nine genes, we constructed a prediction model for OA, which can be utilized for diagnosis and treatment purposes. Previous studies have confirmed that sirtuins (SIRT) play a crucial role in the regulation of cellular metabolism and are associated with various diseases. SIRT4, a member of the SIRT protein family, is specifically localized within mitochondria and maintains mitochondrial function and homeostasis (30). Overexpression of SIRT4 contributes to the inhibition of inflammation and oxidative stress in OA (31). SIRT4 shows a positive correlation with the transcriptional activity of hypoxia-inducible factor-2α (HIF-2α) in chondrocytes. HIF-2α transcription upregulates NAMPT in articular chondrocytes, leading to the development of OA in mice. The interplay between HIF-2α and the NAMPT-NAD(+)-SIRT axis in chondrocytes is associated with HIF-2α or NAMPT-induced cartilage destruction in OA (32). Overexpression of SIRT4 in chondrocytes enhances mitochondrial autophagy, preventing cellular senescence and degradation (33). DNAJC15, also known as methylation-controlled J protein (MCJ), is an inner mitochondrial membrane protein serving as an endogenous inhibitor of respiratory chain complex I. Its depletion results in enhanced mitochondrial respiration and elevated reactive oxygen species levels (34). It is part of the mitochondrial protein transport mechanism and is involved in cell death processes (35). Lack of MCJ affects the pathological physiology resulting from alterations in mitochondrial metabolism (36). NFS1 (cysteine desulfurase) provides persulfide for the biosynthesis of iron-sulfur (FeS) clusters in mitochondria (37). FeS clusters are essential for the activity of many cellular proteins. FKBP8 belongs to the FK506-binding protein (FKBP) family and is a membrane-associated protein typically found in mitochondria. FKBP8 regulates autophagy by interacting with the VPS34 lipid kinase complex (38). Solute carrier family 25 member 37 (SLC25A37), also known as mitochondrial iron transporter-1, is a member of the solute carrier family located in the inner mitochondrial membrane (39). It mediates the uptake of mitochondrial iron. Persulfides are sulfur-containing organic compounds that possess diverse physiological functions. The synthesis of persulfides is primarily mediated by mitochondrial cysteinyl-tRNA synthetase (CARS2). Persulfides mediate mitochondrial biogenesis and bioenergetics. The function of mitochondria in biogenesis and bioenergetics is also supported and upregulated by persulfides derived from mitochondrial cysteinyl-tRNA synthetase (CARS, also known as CARS2) (40). Methylenetetrahydrofolate dehydrogenase 2 (MTHFD2) is a mitochondrial enzyme that participates in one-carbon metabolism. It plays a crucial role in maintaining the integrity of the mitochondrial respiratory chain and preventing mitochondrial dysfunction (41). The electron transfer flavoprotein (ETF), consisting of α and β subunits (ETF α and ETF β, respectively), functions as a mitochondrial matrix protein. It facilitates electron transfer between various mitochondrial dehydrogenases and the primary respiratory chain through the action of electron-transferring flavoprotein dehydrogenase (ETFDH) (42). Pyruvate dehydrogenase kinase 4 (PDK4) inhibits the development of OA through activation of the PPAR pathway (43). Overexpression of PDK4 is also sufficient to promote mitochondrial fission. The PDK4-SEPT2-DRP1 axis has been identified as a regulator of mitochondrial function, bridging the gap between cellular bioenergetics and mitochondrial dynamics (44). However, apart from SIRT4 and PDK4, no other studies have been found examining the remaining seven genes in relation to OA. Our study further confirms the association between mitochondrial metabolism and the progression of OA. Additionally, This study sheds light on the involvement of these mitochondrial metabolism-related genes in the pathophysiological processes of OA, offering valuable insights for the future development of targeted therapeutic strategies for this condition.

We further analyzed the correlation between these nine mitochondrial metabolism genes and hallmark signaling pathways. The results revealed significant associations with various signaling pathways, including unfolded protein response, TNFα signaling via NF-κB, TGF-beta signaling, reactive oxygen species pathway, PI3K-AKT-mTOR signaling, peroxisome, p53 pathway, oxidative phosphorylation, notch signaling, mTORC1 signaling, mitotic spindle, and DNA repair. Mitochondrial unfolded protein response (UPR) is a signaling pathway from mitochondria to the nucleus that is activated to maintain mitochondrial function when misfolded proteins accumulate within the mitochondria. Studies have found that enhanced activation of mitochondrial UPR can reduce chondrocyte death, alleviate joint pain, and lower inflammation levels in synovial fluid (45). TNF-α, a key inflammatory factor in OA pathogenesis, induces chondrocyte death. Research by M J López-Armada et al. demonstrated that TNF-α and interleukin-1 (IL-1) decrease complex I activity and ATP production, ultimately resulting in mitochondrial depolarization and contributing to cartilage degradation (46). TGF-β3, another growth factor present in OA-affected joints, has shown significant potential in promoting chondrocyte growth and metabolism (47). Specifically, TGF-β3 enhances mitochondrial biogenesis by stimulating mitochondrial fission. TGF-β3-induced mitochondrial fission is mediated by the AMPK signaling pathway. ROS leads to chondrocyte apoptosis, reduced synthesis and activation of matrix metalloproteinases, cartilage degradation, and mitochondrial DNA damage (48). Mitochondria-generated ROS contribute significantly to the pathogenesis of OA. In an OA rat model, artemisinin has been found to activate mitochondrial autophagy by inhibiting the PI3K/AKT/mTOR signaling pathway in IL-1β-induced chondrocytes, leading to the amelioration of disease progression (49). Maintaining a normal number and function of mitochondria relies on the crucial process of mitochondrial biogenesis, which is governed by the central regulator peroxisome proliferator-activated receptor γ coactivator-1α (PGC-1α). Sestrin2 (Sesn2), a novel stress-inducible protein, activates AMPK/PGC-1α-mediated mitochondrial biogenesis, thus relieving pain in an OA rat model (50). Curcumin improves OA cartilage degradation by regulating iron death through the P53 signaling pathway (51). Oxidative stress damage can lead to chronic and persistent mitochondrial dysfunction. Oxidative stress is critically involved in the development of certain characteristics of osteoarthritis, including synovial inflammation, cellular senescence and apoptosis, impaired autophagy, and extracellular matrix degradation (52). PGC-1α, a master regulator of mitochondrial biogenesis, plays a protective role in cartilage. PGC-1α delays the onset and progression of OA by influencing mitochondrial biogenesis, oxidative stress, mitochondrial autophagy, and mitochondrial DNA replication in chondrocytes (53). Studies have found that mitochondrial DNA damage is present in chondrocytes of OA patients, accompanied by reduced DNA repair capacity (54). The NOTCH signaling pathway is involved in the metabolic processes of cartilage (55). mTORC1 selectively promotes translation of nuclear-encoded mitochondrial mRNA by inhibiting eukaryotic translation initiation factor 4E (eIF4E) binding protein (4E-BP), thereby controlling mitochondrial activity and biogenesis (56). Thus, mitochondria-related genes may play a role in the development of OA through these signaling pathways mentioned above.

Recent research indicates that alterations in inflammation and the immune system contribute to the pathogenesis of OA (57, 58). Moreover, mitochondrial metabolism plays a pivotal role in the metabolism and activation of immune cells. Consequently, we investigated the relationship between mitochondrial metabolism-related genes and immune cells, identifying significant associations primarily with macrophages, neutrophils, T cells, and dendritic cells. Reprogramming the mitochondrial metabolism of pro-inflammatory M1 macrophages towards an anti-inflammatory M2 phenotype has emerged as a promising strategy for mitigating OA progression. Through modifications in the mitochondrial metabolism of M1 macrophages, repolarization into M2 macrophages can be achieved, effectively suppressing synovial inflammation and impeding early-stage OA progression (59). Neutrophils are the first immune cells to enter the synovium after joint injury, and their activity is an indispensable prerequisite for the progression of OA (60). Research has revealed that neutrophil elastase (NE) induces chondrocyte apoptosis and promotes the development of OA through the cysteine cathepsin signaling pathway (61). NE, the primary inflammatory protease released by neutrophils, is a key factor in OA. Elevated expression levels of various T cell subtypes, including CD4+ and CD8+ T cells, have been observed in OA patients, indicating their potential role in the pathogenesis of the disease (62). Dendritic cells (DCs) in OA patients also exhibit heightened levels of inflammatory cytokines, suggesting their involvement in the pathogenesis of OA (63). While these immune cell types have been implicated in OA pathogenesis, their precise roles in disease initiation and progression remain poorly understood. Therefore, identifying and comprehending the specific immune cell subtypes involved in OA will provide valuable insights into the disease’s etiology and aid in the development of effective treatment strategies.

Inflammation has been shown to play a crucial role in the pathogenesis of OA, particularly through the action of inflammatory cytokines (64). In our study, we identified multiple cytokines that are closely associated with the nine mitochondrial metabolism-related genes. Previous research has reported that interleukin-1β (IL-1β) induces chondrocyte apoptosis by inhibiting SIRT3 expression and mitochondrial autophagy. Mitoquinone (MitoQ5) protects chondrocytes against IL-1β-induced oxidative stress and promotes cell survival by upregulating the SIRT3/Parkin-associated autophagy signaling pathway (65). IL-1β increases mitochondrial iron levels to trigger chondrocyte ferroptosis, leading to inflammatory damage (66). In recent years, many studies have demonstrated that alleviating disease progression in OA can be achieved by inhibiting inflammatory cytokines. Artemisinin (AT) has been reported to activate mitochondrial autophagy and alleviate OA by reducing TNFSF11 expression and inhibiting the PI3K/AKT/mTOR signaling pathway (49). Curcumin (CAD) can mitigate inflammation, cartilage degradation, and ferroptosis induced by IL-1β (51). Nodakenin, in an OA mouse model, reduces chondrocyte degradation and inflammatory responses by inhibiting the expression of inflammatory cytokines such as COX-2, IL-1β, and TNF-α (11). Hydrogen sulfide may counteract IL-1β-induced inflammation and mitochondrial dysfunction-related cellular apoptosis in chondrocytes by inhibiting the PI3K/Akt/NF-κB and MAPK signaling pathways (67).

In recent years, researchers have discovered that miRNAs play important roles in the pathogenesis of OA by mediating processes such as extracellular matrix metabolism, chondrocyte proliferation and apoptosis, oxidative stress, and inflammatory responses (68). Therefore, we analyzed the miRNAs associated with the nine mitochondrial metabolism-related genes (SIRT4, DNAJC15, NFS1, FKBP8, SLC25A37, CARS2, MTHFD2, ETFDH, and PDK4) in OA. Previous studies have found that miR-483-5p promotes extracellular matrix degradation in chondrocytes, thereby accelerating the progression of OA (69). Additionally, in OA cartilage, miR-558 promotes chondrocyte catabolism by targeting COX-2 and regulating IL-1β (70). Upregulated miR-126-5p in OA chondrocytes activates mitochondrial autophagy by downregulating PGC1α expression, leading to chondrocyte degradation and apoptosis (71). Knockdown of miR-222-3p and miR-766-3p exacerbates apoptosis, inflammatory response, extracellular matrix degradation, and oxidative stress in IL-1β-induced OA chondrocytes (72, 73). Downregulation of miR-214-3p accelerates ECM metabolism and apoptosis in chondrocytes by activating the NF-κB signaling pathway, thereby promoting OA development (74). The circFNDC3B/miR-525-5p/HO-1 signaling pathway may alleviate extracellular matrix degradation in OA chondrocytes by mitigating oxidative stress and modulating the NF-κB pathway (75). These findings highlight the potential of miRNAs as biomarkers for evaluating disease progression and prognosis in OA.

In our study, we acknowledge that there are certain limitations that need to be addressed. Firstly, although we utilized publicly available data for analysis, the reliability of the data could be a potential concern. While efforts were made to ensure data quality and consistency, it is important to note that the original data sources were not generated specifically for our study. Further validation using independent datasets or experimental verification would enhance the robustness of our findings. Secondly, while our pre-modeling approach showed promising results, clinical validation is required to establish the utility and accuracy of the predictive model we developed. Incorporating clinical data and conducting prospective studies would provide valuable insights into the real-world applicability and performance of the model. Lastly, as this study focused on bioinformatics analysis, it is essential to clearly define the directions for future experimental studies. Identifying key genes and pathways associated with mitochondria in osteoarthritis opens up avenues for experimental validation, functional characterization, and mechanistic investigations. Future experimental studies should aim to elucidate the biological relevance of these genes, explore their interactions within cellular processes, and investigate potential therapeutic targets.

In conclusion, the involvement of mitochondrial metabolism in the onset and progression of OA is significant. Targeting mitochondrial dysfunction presents a promising therapeutic approach for OA cases associated with impaired mitochondrial function. However, there is currently a lack of identified drugs that specifically target mitochondrial metabolism in OA. Further research is necessary to comprehend the complex interplay between mitochondrial metabolism and OA. Mitochondrial-targeted therapies hold potential in providing effective preventive and treatment strategies for OA.

## Data availability statement

The original contributions presented in the study are included in the article/**Supplementary Material**. Further inquiries can be directed to the corresponding authors.

## Author contributions

YW: Writing – original draft, Visualization, Validation, Methodology, Data curation. HH: Writing – review & editing. TW: Writing – original draft, Validation, Methodology. WG: Writing – original draft, Methodology. SZ: Writing – review & editing, Supervision, Methodology. RW: Writing – review & editing, Supervision, Resources, Methodology, Funding acquisition.

## References

[1] GBD 2019 Diseases and Injuries Collaborators. Global burden of 369 diseases and injuries in 204 countries and territories, 1990-2019: a systematic analysis for the Global Burden of Disease Study 2019 [published correction appears in Lancet. 2020 Nov 14;396(10262):1562. doi: 10.1016/S0140-6736(20)32226-1]. Lancet. (2020) 396(10258):1204–22. doi: 10.1016/S0140-6736(20)30925-9IF PMC7567026 33069326

[2] Chen CL Lin CY Kung HJ. Targeting mitochondrial OXPHOS and their regulatory signals in prostate cancers. Int J Mol Sci. (2021) 22(24):13435. doi: 10.3390/ijms222413435 34948229 PMC8708687

[3] Rong Z Tu P Xu P Sun Y Yu F Tu N. The mitochondrial response to DNA damage. Front Cell Dev Biol. (2021) 9:669379. doi: 10.3389/fcell.2021.669379 34055802 PMC8149749

[4] Sun K Jing X Guo J Yao X Guo F. Mitophagy in degenerative joint diseases. Autophagy. (2021) 17:2082–92. doi: 10.1080/15548627.2020.1822097 PMC8496714 32967533

[5] Kumar P Liu C Hsu JW Chacko S Minard C Jahoor F. Glycine and N-acetylcysteine (GlyNAC) supplementation in older adults improves glutathione deficiency, oxidative stress, mitochondrial dysfunction, inflammation, insulin resistance, endothelial dysfunction, genotoxicity, muscle strength, and cognition: Results of a pilot clinical trial. Clin Trans Med. (2021) 11:e372. doi: 10.1002/ctm2.372 PMC8002905 33783984

[6] Hosseinzadeh A Kamrava SK Joghataei MT Darabi R Shakeri-Zadeh A Shahriari M. Apoptosis signaling pathways in osteoarthritis and possible protective role of melatonin. J pineal Res. (2016) 61:411–25. doi: 10.1111/jpi.12362 27555371

[7] Ansari MY Novak K Haqqi TM. ERK1/2-mediated activation of DRP1 regulates mitochondrial dynamics and apoptosis in chondrocytes. Osteoarthritis cartilage. (2022) 30:315–28. doi: 10.1016/j.joca.2021.11.003 PMC8792336 34767958

[8] Zheng L Zhang Z Sheng P Mobasheri A. The role of metabolism in chondrocyte dysfunction and the progression of osteoarthritis. Ageing Res Rev. (2021) 66:101249. doi: 10.1016/j.arr.2020.101249 33383189

[9] Liu D Cai ZJ Yang YT Lu WH Pan LY Xiao WF. Mitochondrial quality control in cartilage damage and osteoarthritis: new insights and potential therapeutic targets. Osteoarthritis cartilage. (2022) 30:395–405. doi: 10.1016/j.joca.2021.10.009 34715366

[10] Wang Y Liu HH Cao YT Zhang LL Huang F Yi C. The role of mitochondrial dynamics and mitophagy in carcinogenesis, metastasis and therapy. Front Cell Dev Biol. (2020) 8:413. doi: 10.3389/fcell.2020.00413 32587855 PMC7297908

[11] Yi N Mi Y Xu X Li N Chen B Yan K. Nodakenin attenuates cartilage degradation and inflammatory responses in a mice model of knee osteoarthritis by regulating mitochondrial Drp1/ROS/NLRP3 axis. Int Immunopharmacol. (2022) 113:109349. doi: 10.1016/j.intimp.2022.109349 36302323

[12] Zeng Z Zhou X Wang Y Cao H Guo J Wang P. Mitophagy-a new target of bone disease. Biomolecules (2022) 12(10):1420. doi: 10.3390/biom12101420 36291629 PMC9599755

[13] Parker HS Leek JT Favorov AV Considine M Xia X Chavan S. Preserving biological heterogeneity with a permuted surrogate variable analysis for genomics batch correction. Bioinf (Oxford England). (2014) 30:2757–63. doi: 10.1093/bioinformatics/btu375 PMC4173013 24907368

[14] Zhang B Sun J Guan H Guo H Huang B Chen X. Integrated single-cell and bulk RNA sequencing revealed the molecular characteristics and prognostic roles of neutrophils in pancreatic cancer. Aging. (2023) 15:9718–42. doi: 10.18632/aging.v15i18 PMC10564426 37728418

[15] Chi H Jiang P Xu K Zhao Y Song B Peng G. A novel anoikis-related gene signature predicts prognosis in patients with head and neck squamous cell carcinoma and reveals immune infiltration. Front Genet. (2022) 13:984273. doi: 10.3389/fgene.2022.984273 36092898 PMC9459093

[16] Zhao S Chi H Yang Q Chen S Wu C Lai G. Identification and validation of neurotrophic factor-related gene signatures in glioblastoma and Parkinson’s disease. Front Immunol. (2023) 14:1090040. doi: 10.3389/fimmu.2023.1090040 36825022 PMC9941742

[17] Chi H Peng G Yang J Zhang J Song G Xie X. Machine learning to construct sphingolipid metabolism genes signature to characterize the immune landscape and prognosis of patients with uveal melanoma. Front endocrinology. (2022) 13:1056310. doi: 10.3389/fendo.2022.1056310 PMC9772281 36568076

[18] Chen Y Li C Wang N Wu Z Zhang J Yan J. Identification of LINC00654-NINL regulatory axis in diffuse large B-cell lymphoma in silico analysis. Front Oncol. (2022) 12:883301. doi: 10.3389/fonc.2022.883301 35719990 PMC9204339

[19] Chi H Peng G Wang R Yang F Xie X Zhang J. Cuprotosis programmed-cell-death-related lncRNA signature predicts prognosis and immune landscape in PAAD patients. Cells. (2022) 11. doi: 10.3390/cells11213436 PMC9658590 36359832

[20] Chi H Yang J Peng G Zhang J Song G Xie X. Circadian rhythm-related genes index: A predictor for HNSCC prognosis, immunotherapy efficacy, and chemosensitivity. Front Immunol. (2023) 14:1091218. doi: 10.3389/fimmu.2023.1091218 36969232 PMC10036372

[21] Wu X Lu W Xu C Jiang C Zhuo Z Wang R. Macrophages phenotype regulated by IL-6 are associated with the prognosis of platinum-resistant serous ovarian cancer: integrated analysis of clinical trial and omics. J Immunol Res. (2023) 2023:6455704. doi: 10.1155/2023/6455704 37124547 PMC10132904

[22] Xu W Li H Hameed Y Abdel-Maksoud MA Almutairi SM Mubarak A. Elucidating the clinical and immunological value of m6A regulator-mediated methylation modification patterns in adrenocortical carcinoma. Oncol Res. (2023) 31:819–31. doi: 10.32604/or.2023.029414 PMC10398396 37547754

[23] Yu G Wang LG Han Y He QY. clusterProfiler: an R package for comparing biological themes among gene clusters. Omics: J Integr Biol. (2012) 16:284–7. doi: 10.1089/omi.2011.0118 PMC3339379 22455463

[24] Newman AM Liu CL Green MR Gentles AJ Feng W Xu Y. Robust enumeration of cell subsets from tissue expression profiles. Nat Methods. (2015) 12:453–7. doi: 10.1038/nmeth.3337 PMC4739640 25822800

[25] Piñero J Saüch J Sanz F Furlong LI. The DisGeNET cytoscape app: Exploring and visualizing disease genomics data. Comput Struct Biotechnol J. (2021) 19:2960–7. doi: 10.1016/j.csbj.2021.05.015 PMC8163863 34136095

[26] Lock EF Dunson DB. Bayesian consensus clustering. Bioinf (Oxford England). (2013) 29:2610–6. doi: 10.1093/bioinformatics/btt425 PMC3789539 23990412

[27] Zhao S Zhang X Gao F Chi H Zhang J Xia Z. Identification of copper metabolism-related subtypes and establishment of the prognostic model in ovarian cancer. Front endocrinology. (2023) 14:1145797. doi: 10.3389/fendo.2023.1145797 PMC10025496 36950684

[28] Yuan Q Lu X Guo H Sun J Yang M Liu Q. Low-density lipoprotein receptor promotes crosstalk between cell stemness and tumor immune microenvironment in breast cancer: a large data-based multi-omics study. J Trans Med. (2023) 21:871. doi: 10.1186/s12967-023-04699-y PMC10691045 38037058

[29] Blanco FJ Rego I Ruiz-Romero C. The role of mitochondria in osteoarthritis. Nat Rev Rheumatol. (2011) 7:161–9. doi: 10.1038/nrrheum.2010.213 21200395

[30] Ji Z Liu GH Qu J. Mitochondrial sirtuins, metabolism, and aging. J Genet Genomics = Yi Chuan xue bao. (2022) 49:287–98. doi: 10.1016/j.jgg.2021.11.005 34856390

[31] Dai Y Liu S Li J Li J Lan Y Nie H. SIRT4 suppresses the inflammatory response and oxidative stress in osteoarthritis. Am J Trans Res. (2020) 12:1965–75. PMC7270038 32509191

[32] Oh H Kwak JS Yang S Gong MK Kim JH Rhee J. Reciprocal regulation by hypoxia-inducible factor-2α and the NAMPT-NAD(+)-SIRT axis in articular chondrocytes is involved in osteoarthritis. Osteoarthritis cartilage. (2015) 23:2288–96. doi: 10.1016/j.joca.2015.07.009 26209889

[33] Lin S Wu B Hu X Lu H. Sirtuin 4 (Sirt4) downregulation contributes to chondrocyte senescence and osteoarthritis via mediating mitochondrial dysfunction. Int J Biol Sci. (2024) 20:1256–78. doi: 10.7150/ijbs.85585 PMC10878156 38385071

[34] Navasa N Martín I Iglesias-Pedraz JM Beraza N Atondo E Izadi H. Regulation of oxidative stress by methylation-controlled J protein controls macrophage responses to inflammatory insults. J Infect diseases. (2015) 211:135–45. doi: 10.1093/infdis/jiu389 PMC4326314 25028693

[35] Sinha D D’Silva P. Chaperoning mitochondrial permeability transition: regulation of transition pore complex by a J-protein, DnaJC15. Cell Death disease. (2014) 5:e1101. doi: 10.1038/cddis.2014.72 24603329 PMC3973195

[36] Hatle KM Gummadidala P Navasa N Bernardo E Dodge J Silverstrim B. MCJ/DnaJC15, an endogenous mitochondrial repressor of the respiratory chain that controls metabolic alterations. Mol Cell Biol. (2013) 33:2302–14. doi: 10.1128/MCB.00189-13 PMC3648061 23530063

[37] Uzarska MA Grochowina I Soldek J Jelen M Schilke B Marszalek J. During FeS cluster biogenesis, ferredoxin and frataxin use overlapping binding sites on yeast cysteine desulfurase Nfs1. J Biol Chem. (2022) 298:101570. doi: 10.1016/j.jbc.2022.101570 35026224 PMC8888459

[38] Aguilera MO Robledo E Melani M Wappner P Colombo MI. FKBP8 is a novel molecule that participates in the regulation of the autophagic pathway. Biochim Biophys Acta Mol Cell Res. (2022) 1869:119212. doi: 10.1016/j.bbamcr.2022.119212 35090967

[39] Chen W Paradkar PN Li L Pierce EL Langer NB Takahashi-Makise N. Abcb10 physically interacts with mitoferrin-1 (Slc25a37) to enhance its stability and function in the erythroid mitochondria. Proc Natl Acad Sci United States America. (2009) 106:16263–8. doi: 10.1073/pnas.0904519106 PMC2752562 19805291

[40] Fujii S Sawa T Motohashi H Akaike T. Persulfide synthases that are functionally coupled with translation mediate sulfur respiration in mammalian cells. Br J Pharmacol. (2019) 176:607–15. doi: 10.1111/bph.14356 PMC6346073 29748969

[41] Yue L Pei Y Zhong L Yang H Wang Y Zhang W. Mthfd2 modulates mitochondrial function and DNA repair to maintain the pluripotency of mouse stem cells. Stem Cell Rep. (2020) 15:529–45. doi: 10.1016/j.stemcr.2020.06.018 PMC7419720 32679066

[42] Purevjav E Kimura M Takusa Y Ohura T Tsuchiya M Hara N. Molecular study of electron transfer flavoprotein alpha-subunit deficiency in two Japanese children with different phenotypes of glutaric acidemia type II. Eur J Clin Invest. (2002) 32:707–12. doi: 10.1046/j.1365-2362.2002.01045.x 12486872

[43] Li Z Xie L Zeng H Wu Y. PDK4 inhibits osteoarthritis progression by activating the PPAR pathway. J orthopaedic Surg Res. (2024) 19:109. doi: 10.1186/s13018-024-04583-5 PMC10835968 38308345

[44] Thoudam T Chanda D Sinam IS Kim BG Kim MJ Oh CJ. Noncanonical PDK4 action alters mitochondrial dynamics to affect the cellular respiratory status. Proc Natl Acad Sci United States America. (2022) 119:e2120157119. doi: 10.1073/pnas.2120157119 PMC9407676 35969774

[45] Zhou Z Lu J Yang M Cai J Fu Q Ma J. The mitochondrial unfolded protein response (UPR(mt)) protects against osteoarthritis. Exp Mol Med. (2022) 54:1979–90. doi: 10.1038/s12276-022-00885-y PMC9723171 36380018

[46] López-Armada MJ Caramés B Martín MA Cillero-Pastor B Lires-Dean M Fuentes-Boquete I. Mitochondrial activity is modulated by TNFalpha and IL-1beta in normal human chondrocyte cells. Osteoarthritis cartilage. (2006) 14:1011–22. 10.1016/j.joca.2006.03.008 16679036

[47] Du X Duan M Kan S Yang Y Xu S Wei J. TGF-β3 mediates mitochondrial dynamics through the p-Smad3/AMPK pathway. Cell Prolif. (2024) 57(5):e13579. doi: 10.1111/cpr.13579IF 38012096 PMC11056712

[48] Hou L Wang G Zhang X Lu F Xu J Guo Z. Mitoquinone alleviates osteoarthritis progress by activating the NRF2-Parkin axis. iScience. (2023) 26:107647. doi: 10.1016/j.isci.2023.107647 37694150 PMC10483061

[49] Li J Jiang M Yu Z Xiong C Pan J Cai Z. Artemisinin relieves osteoarthritis by activating mitochondrial autophagy through reducing TNFSF11 expression and inhibiting PI3K/AKT/mTOR signaling in cartilage. Cell Mol Biol letters. (2022) 27:62. doi: 10.1186/s11658-022-00365-1 PMC9331798 35902802

[50] Sargeant TJ Fourrier C. Human monocyte-derived microglia-like cell models: A review of the benefits, limitations and recommendations. Brain behavior immunity. (2023) 107:98–109. doi: 10.1016/j.bbi.2022.09.015 36202170

[51] Gong Z Wang Y Li L Li X Qiu B Hu Y. Cardamonin alleviates chondrocytes inflammation and cartilage degradation of osteoarthritis by inhibiting ferroptosis via p53 pathway. Food Chem toxicology: an Int J published Br Ind Biol Res Assoc. (2023) 174:113644. doi: 10.1016/j.fct.2023.113644 36731815

[52] Jiang W Liu H Wan R Wu Y Shi Z Huang W. Mechanisms linking mitochondrial mechanotransduction and chondrocyte biology in the pathogenesis of osteoarthritis. Ageing Res Rev. (2021) 67:101315. doi: 10.1016/j.arr.2021.101315 33684550

[53] Wang H Su J Yu M Xia Y Wei Y. PGC-1α in osteoarthritic chondrocytes: From mechanism to target of action. Front Pharmacol. (2023) 14:1169019. doi: 10.3389/fphar.2023.1169019 37089944 PMC10117990

[54] Grishko VI Ho R Wilson GL Pearsall A. Diminished mitochondrial DNA integrity and repair capacity in OA chondrocytes. Osteoarthritis cartilage. (2009) 17:107–13. doi: 10.1016/j.joca.2008.05.009 PMC3640312 18562218

[55] Liu Z Chen J Mirando AJ Wang C Zuscik MJ O’Keefe RJ. A dual role for NOTCH signaling in joint cartilage maintenance and osteoarthritis. Sci Signaling. (2015) 8:ra71. doi: 10.1126/scisignal.aaa3792 PMC4607068 26198357

[56] Davis OB Shin HR Lim CY Wu EY Kukurugya M Maher CF. NPC1-mTORC1 signaling couples cholesterol sensing to organelle homeostasis and is a targetable pathway in niemann-pick type C. Dev Cell. (2021) 56:260–76.e7. doi: 10.1016/j.devcel.2020.11.016 33308480 PMC8919971

[57] Woodell-May JE Sommerfeld SD. Role of inflammation and the immune system in the progression of osteoarthritis. J orthopaedic research: Off Publ Orthopaedic Res Society. (2020) 38:253–7. doi: 10.1002/jor.24457 31469192

[58] Chow YY Chin KY. The role of inflammation in the pathogenesis of osteoarthritis. Mediators inflammation. (2020) 2020:8293921. doi: 10.1155/2020/8293921 PMC7072120 32189997

[59] Zhang L Chen X Cai P Sun H Shen S Guo B. Reprogramming mitochondrial metabolism in synovial macrophages of early osteoarthritis by a camouflaged meta-defensome. Advanced materials (Deerfield Beach Fla). (2022) 34:e2202715. doi: 10.1002/adma.202202715 35671349

[60] Chaney S Vergara R Qiryaqoz Z Suggs K Akkouch A. The involvement of neutrophils in the pathophysiology and treatment of osteoarthritis. Biomedicines. (2022) 10. doi: 10.3390/biomedicines10071604 PMC9313259 35884909

[61] Wang G Jing W Bi Y Li Y Ma L Yang H. Neutrophil elastase induces chondrocyte apoptosis and facilitates the occurrence of osteoarthritis via caspase signaling pathway. Front Pharmacol. (2021) 12:666162. doi: 10.3389/fphar.2021.666162 33935789 PMC8080035

[62] Zhu W Zhang X Jiang Y Liu X Huang L Wei Q. Alterations in peripheral T cell and B cell subsets in patients with osteoarthritis. Clin Rheumatol. (2020) 39:523–32. doi: 10.1007/s10067-019-04768-y 31624962

[63] Alahdal M Zhang H Huang R Sun W Deng Z Duan L. Potential efficacy of dendritic cell immunomodulation in the treatment of osteoarthritis. Rheumatol (Oxford England). (2021) 60:507–17. doi: 10.1093/rheumatology/keaa745 33249512

[64] Motta F Barone E Sica A Selmi C. Inflammaging and osteoarthritis. Clin Rev Allergy Immunol. (2023) 64:222–38. doi: 10.1007/s12016-022-08941-1 35716253

[65] Xin R Xu Y Long D Mao G Liao H Zhang Z. Mitochonic acid-5 inhibits reactive oxygen species production and improves human chondrocyte survival by upregulating SIRT3-mediated, parkin-dependent mitophagy. Front Pharmacol. (2022) 13:911716. doi: 10.3389/fphar.2022.911716 35734404 PMC9207248

[66] Wang X Liu Z Peng P Gong Z Huang J Peng H. Astaxanthin attenuates osteoarthritis progression via inhibiting ferroptosis and regulating mitochondrial function in chondrocytes. Chemico-biological interactions. (2022) 366:110148. doi: 10.1016/j.cbi.2022.110148 36084724

[67] Wang B Shao Z Gu M Ni L Shi Y Yan Y. Hydrogen sulfide protects against IL-1β-induced inflammation and mitochondrial dysfunction-related apoptosis in chondrocytes and ameliorates osteoarthritis. J Cell Physiol. (2021) 236:4369–86. doi: 10.1002/jcp.30154 33164235

[68] Al-Modawi RN Brinchmann JE Karlsen TA. Multi-pathway protective effects of microRNAs on human chondrocytes in an *in vitro* model of osteoarthritis. Mol Ther Nucleic Acids. (2019) 17:776–90. doi: 10.1016/j.omtn.2019.07.011 PMC6716067 31446120

[69] Wang H Zhang H Sun Q Wang Y Yang J Yang J. Intra-articular delivery of antago-miR-483-5p inhibits osteoarthritis by modulating matrilin 3 and tissue inhibitor of metalloproteinase 2. Mol therapy: J Am Soc Gene Ther. (2017) 25:715–27. doi: 10.1016/j.ymthe.2016.12.020 PMC5363189 28139355

[70] Park SJ Cheon EJ Kim HA. MicroRNA-558 regulates the expression of cyclooxygenase-2 and IL-1β-induced catabolic effects in human articular chondrocytes. Osteoarthritis cartilage. (2013) 21:981–9. doi: 10.1016/j.joca.2013.04.012 23611898

[71] Kim D Song J Jin EJ. BNIP3-dependent mitophagy via PGC1α Promotes cartilage degradation. Cells. (2021) 10. doi: 10.3390/cells10071839 PMC8304751 34360007

[72] Lu J Wu W. Cholinergic modulation of the immune system - A novel therapeutic target for myocardial inflammation. Int immunopharmacology. (2021) 93:107391. doi: 10.1016/j.intimp.2021.107391 33548577

[73] Li Z Cheng J Liu J. Baicalin protects human OA chondrocytes against IL-1β-induced apoptosis and ECM degradation by activating autophagy via miR-766-3p/AIFM1 axis. Drug design Dev Ther. (2020) 14:2645–55. doi: 10.2147/DDDT.S255823 PMC7353997 32753846

[74] Cao Y Tang S Nie X Zhou Z Ruan G Han W. Decreased miR-214-3p activates NF-κB pathway and aggravates osteoarthritis progression. EBioMedicine. (2021) 65:103283. doi: 10.1016/j.ebiom.2021.103283 33714889 PMC7957119

[75] Chen Z Huang Y Chen Y Yang X Zhu J Xu G. CircFNDC3B regulates osteoarthritis and oxidative stress by targeting miR-525-5p/HO-1 axis. Commun Biol. (2023) 6:200. doi: 10.1038/s42003-023-04569-9 36806251 PMC9941484

